# Single‐Cell RNA Sequencing of Retina Reveals Nna1 Upregulation in Myopic Diabetic Retinopathy as a Protective Factor Against Diabetic Damage

**DOI:** 10.1002/advs.202500438

**Published:** 2025-11-05

**Authors:** Lihui Xie, Yingjun Cai, Bolin Chen, Bowen Li, Jing Zou, Huizhuo Xu

**Affiliations:** ^1^ Eye Center of Xiangya Hospital Hunan Key Laboratory of Ophthalmology Central South University Changsha Hunan 410008 China; ^2^ National Clinical Research Center for Geriatric Disorders Xiangya Hospital Central South University Changsha Hunan 410008 China

**Keywords:** autophagy, diabetic retinopathy, Müller cell, myopia, Nna1/Agtpbp1

## Abstract

Epidemiological and clinical observations suggest a substantial reduction in the risk and severity of diabetic retinopathy (DR) among individuals with myopia. However, the impact of myopia on DR and its underlying mechanisms remains unclear. Herein, by establishing a form‐deprivation myopia (FDM) and lens‐induced myopia (LIM) models in diabetic db/db mice, a significant reduction of retinal vascular lesions is identified in db/db mice after myopia modeling. Single‐cell transcriptomic analysis further reveals elevated expression of Nervous system nuclear protein induced by axotomy 1 (Nna1) in Müller cells of db/db mice subjected to FDM compared to db/db alone, alongside decreased Nna1 expression in db/db compared to db/m mice. Knockdown of Nna1 in FDM‐treated db/db eyes reverses the protective effects of myopia on DR. Transcriptomic profiling links reduced Nna1 expression to enhanced apoptotic and autophagic signaling pathways in Müller cells. Further in vivo and in vitro experiments confirm that Nna1 overexpression suppresses microtubule hyper‐glutamylation, thereby reducing autophagy and apoptosis levels in Müller cells, and ameliorating DR progression. These findings suggest that Nna1 may play a key role in protecting Müller cells and maintaining the integrity of the neurovascular unit, thereby contributing to the protective effects of myopia in DR and representing a potential molecular target for early intervention and treatment of DR.

## Graphical abstract

**This study investigates a schematic overview of the mechanistic pathways**. The relationship between myopia and DR has long been of clinical interest. In diabetic mice, Nna1 expression is downregulated, whereas in diabetic mice with FDM, Nna1 expression is upregulated–particularly in Müller cells–accompanied by decreased vascular endothelial growth factor (VEGF) expression, reduced vascular leakage, a lower endothelial‐to‐pericyte ratio, and fewer microaneurysms. Overexpression of Nna1 is found to inhibit diabetic retinal neurovascular unit damage by regulating autophagy and apoptosis in Müller cells through the inhibition of tubulin hyper‐glutamylation.

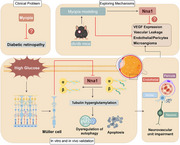

## 1 Introduction

Diabetic retinopathy (DR) is the most prevalent complication of diabetes and a leading cause of blindness among the global working‐age population.^[^ ^1^ ^]^ In 2020, there were ≈100 million DR patients worldwide, with projections expecting this number to increase to 160 million by 2045.^[^ ^2^ ^]^ Based on disease severity, DR is clinically classified into non‐proliferative diabetic retinopathy (NPDR) and proliferative diabetic retinopathy (PDR).^[^ ^1^ ^]^ PDR is characterized by pathological neovascularization originating from the retinal or optic disc vasculature, which may lead to severe complications such as vitreous hemorrhage, tractional retinal detachment, and neovascular glaucoma.^[^ ^1^ ^]^ Both NPDR and PDR can cause varying degrees of visual impairment, especially when the macula is involved. Current treatment strategies include panretinal photocoagulation, and intravitreal injections of anti‐vascular endothelial growth factor (VEGF) agents to suppress neovascularization.^[^ ^3^, ^4^ ^]^ In more severe cases, pars plana vitrectomy is required to manage complications such as persistent vitreous hemorrhage.^[^ ^3^ ^]^ However, these interventions are often associated with suboptimal outcomes and various side effects, highlighting the urgent need to explore novel pathogenic mechanisms and therapeutic targets for DR.

Earlier epidemiological research have shown that the likelihood of developing DR rises with the length of diabetes, inadequate glycemic control, elevated blood pressure, and blood lipids.^[^ ^1^, ^5^, ^6^ ^]^ Additionally, it has been suggested that ocular influences like myopia,^[^ ^7^ ^]^ intraocular pressure,^[^ ^8^ ^]^ and posterior vitreous detachment^[^ ^9^ ^]^ are associated with the development of DR. The idea that myopia, especially high myopia, may act as a protective factor against DR is not new, with Jain et al. potentially first reporting it as early as 1965.^[^ ^10^ ^]^ Since then, a growing body of population‐based and clinical studies has consistently demonstrated that myopia is associated with a reduced incidence and slower progression of both NPDR and PDR in adults.^[^ ^11^, ^12^, ^13^, ^14^, ^15^, ^16^, ^17^ ^]^ Recent meta‐analysis by Fu et al.^[^ ^18^ ^]^ and Wang et al.^[^ ^7^ ^]^ examined the relationship between axial length, refractive error, and DR. Both analyses found that a longer axial length and higher degree of myopic refractive errors are associated with a lower risk of developing DR. Several mechanisms have been proposed to explain this association. First, axial elongation in high myopia mechanically stretches and thins the retina and choroid, leading to narrowing of the retinal vasculature and reduced blood flow. This vascular attenuation lowers the hydrostatic pressure on retinal capillaries, decreasing the risk of leakage and hemorrhage commonly seen in DR.^[^ ^19^, ^20^, ^21^, ^22^ ^]^ Second, outer retinal thinning resulting from degenerative or stretch‐induced changes in myopia—particularly involving photoreceptors in the outer nuclear layer (ONL), which are among the most metabolically active retinal cells and prone to loss or compaction—leads to decreased oxygen consumption, thereby alleviating retinal hypoxia and subsequently suppressing the expression of VEGF and pro‐inflammatory cytokines.^[^ ^20^, ^23^ ^]^ This mechanism is conceptually similar to panretinal photocoagulation, where the ablation of photoreceptors serves to reduce metabolic demand and redirect oxygen to ischemic regions of the retina.^[^ ^24^ ^]^ Third, the presence of posterior vitreous detachment in myopic eyes may facilitate oxygen diffusion through the liquefied vitreous, thereby improving retinal oxygenation.^[^ ^25^ ^]^ Additionally, the elongated axial length and larger intraocular volume in myopic eyes may dilute intraocular VEGF concentrations, particularly in the anterior chamber and vitreous cavity, contributing to a reduced risk of neovascularization and progression to DR.^[^ ^26^ ^]^ Furthermore, high myopia‐induced choroidal thinning may increase the perfusion‐to‐metabolism ratio, further contributing to a reduced retinal metabolic burden.^[^ ^27^ ^]^ Therefore, retinal thinning in high myopia represents a pathological consequence; however, this outcome may paradoxically mitigate DR progression by reducing both metabolic demand and pathological neovascular stimuli.

Though previous research has investigated the potential protective effect of myopia on DR, the majority of these studies relied on clinical studies, meta‐analysis, and population‐based studies. Few have directly examined the underlying mechanisms involved. We therefore conducted this study in diabetic combined with form‐deprivation myopia (FDM) and lens‐induced myopia (LIM) model to 1) confirm the protective role of myopia on DR using a diabetic and myopia mouse model, 2) evaluate the relationship and variations in retinal cell clusters between diabetic mice and those with diabetes combined with myopia, 3) investigate the fundamental impact of possible protective factors and mechanisms in myopia that mitigate DR.

## 2 Results

### 2.1 Establishing a Mouse Model of Diabetes Complicated with Myopia

**Figure 1:**
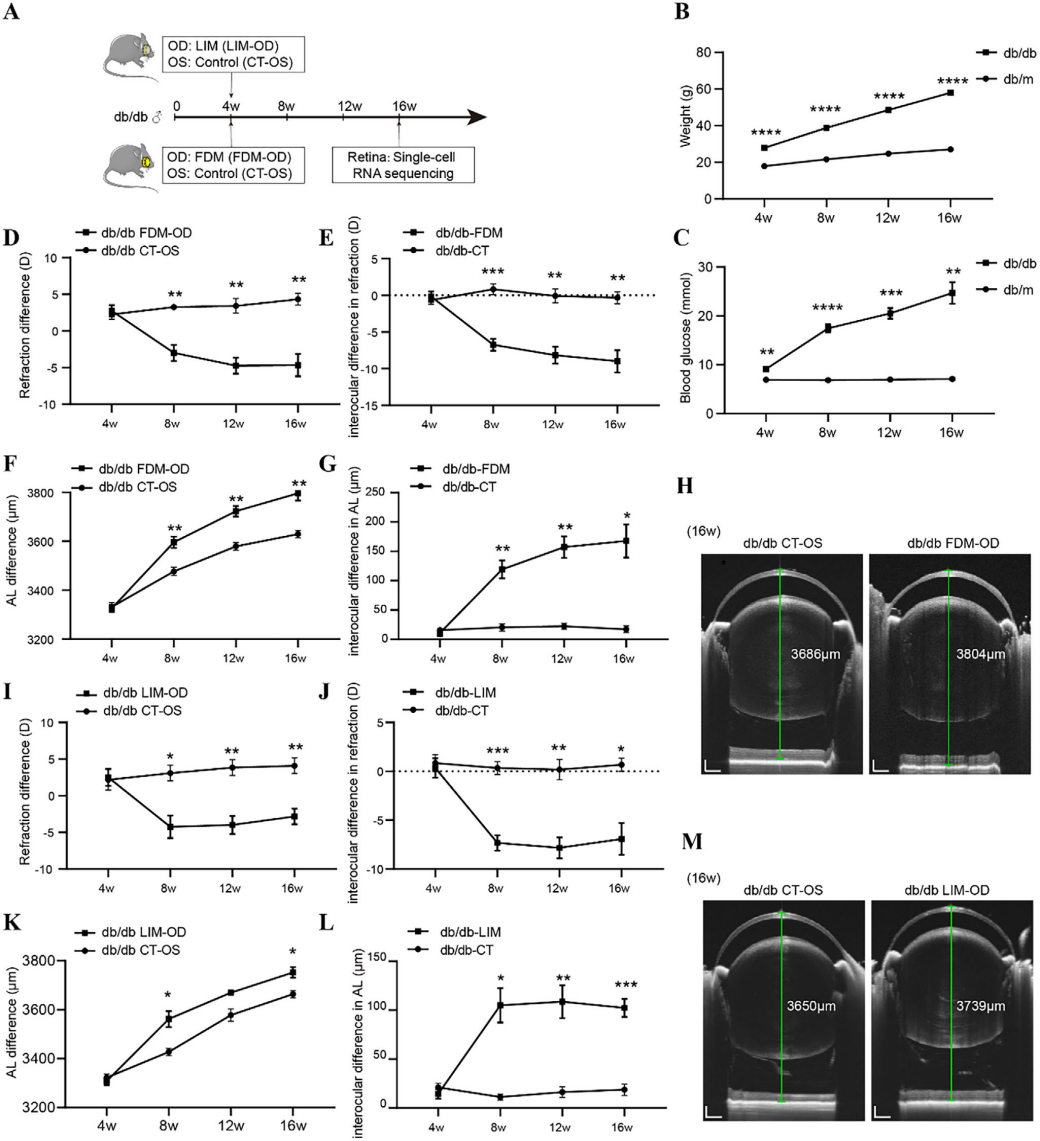
Study design and establishment of a diabetic mouse model combined with myopia (FDM or LIM). A) Schematic of the experimental design: Four‐week‐old db/db mice were utilized to establish a FDM model in right eye (FDM‐OD) or a LIM model in right eye (LIM‐OD), with the left eye serving as the control (CT‐OS). Retina cells were harvested for scRNA‐seq analysis from FDM‐OD or CT‐OS after 12 weeks (16‐week‐old). B,C) Time‐course of body weight (B) and blood glucose (C) levels in db/db and db/m mice. (*n* = 6/group). D) Longitudinal measurements of refraction in db/db CT‐OS and FDM‐OD eyes at 4 weeks of age (baseline) and at 8, 12, and 16 weeks (i.e., 4, 8, and 12 weeks post‐FDM induction). (*n* = 6/group). E) Longitudinal measurements of interocular differences in refraction between db/db CT‐OS and FDM‐OD eyes (db/db‐FDM) and between db/db right and left eyes without myopia induction (db/db‐CT) across time. (*n* = 6/group). F) Longitudinal measurements of axial length in db/db CT‐OS and FDM‐OD eyes across time. (*n* = 6/group). G) Longitudinal measurements of interocular differences in axial length of db/db‐FDM and db/db‐CT groups across time. (*n* = 6/group). H) Representative OCT images of axial length at 16 weeks in db/db CT‐OS and FDM‐OD eyes measured by global‐swept‐source OCT. Scale bars indicate 200 µm. I) Longitudinal measurements of refraction in db/db CT‐OS and LIM‐OD eyes across time. (*n* = 6/group). J) Longitudinal measurements of interocular differences in refraction between db/db CT‐OS and LIM‐OD eyes (db/db‐LIM) and between db/db right and left eyes without myopia induction (db/db‐CT) across time. (*n* = 6/group). K) Longitudinal measurements of axial length in db/db CT‐OS and LIM‐OD eyes across time. (*n* = 6/group). L) Longitudinal measurements of interocular differences in axial length of db/db‐LIM and db/db‐CT groups across time. (*n* = 6/group). M) Representative OCT images of axial length at 16 weeks in db/db CT‐OS and LIM‐OD eyes. Scale bars indicate 200 µm. Results expressed as mean ± SEM. ^*^ *p* <0.05, ^**^ *p* <0.01, ^***^ *p* <0.001, ^****^ *p* <0.0001. *P* values were determined by Repeated Measures ANOVA (B–G, I–L).

To establish a mouse model of diabetes complicated with myopia, we utilized 4‐week‐old C57BL/KsJ‐Lepr^db/db^ spontaneous type II diabetic mice (db/db) and nondiabetic heterozygous littermates (db/m) (**Figure** **1**A). A FDM model was induced by covering the right eye (FDM‐OD), with the left eye serving as control (CT‐OS), and a LIM model was also induced using a −10 diopter (D) lens, similarly applied to the right eye (LIM‐OD) (Figure 1A; Figure S1A,B, Supporting Information).

The body weight of db/db mice increased progressively from 27.88 ± 0.59 g at 4 weeks to 58.00 ± 0.84 g at 16 weeks, showing a near‐linear trend across 4, 8, 12, and 16 weeks (Figure 1B). In contrast, control db/m mice showed a much slower weight gain, from 17.92 ± 0.34 g at 4 weeks to 27.08 ± 0.27 g at 16 weeks (Figure 1B). Similarly, blood glucose levels in db/db mice elevated steadily from 9.12 ± 0.43 mmol L^−1^ at 4 weeks to 24.68 ± 2.22 mmol L^−1^ at 16 weeks, while db/m mice maintained stable glucose levels between 6.90 ± 0.26 and 7.08 ± 0.32 mmol L^−1^ during the same period (Figure 1C), confirming the successful establishment of a type II diabetic mouse model.

Furthermore, we evaluated the development of myopia by assessing changes in ocular refraction and axial length. At 4 weeks, the baseline refraction in db/db FDM‐OD and CT‐OS was 2.75 ± 0.77 D and 2.25 ± 0.66 D, respectively (Figure 1D). After FDM modeling, FDM‐OD eyes gradually developed myopia, reaching −4.67 ± 1.52 D at 16 weeks (12 weeks after occlusion), while the CT‐OS eyes shifted toward hyperopia, reaching 4.33 ± 0.80 D (Figure 1D). The interocular difference in diopter remained negligible in unoccluded db/db mice (db/db‐CT) at 16 weeks (−0.33 ± 0.80 D), but was markedly increased in FDM‐modeled mice (db/db‐FDM) to −9.00 ± 1.53 D (Figure 1E). Similarly, axial length changes followed the same trend. At baseline (4 weeks), axial length was 3326 ± 14.71 µm in FDM‐OD and 3331 ± 18.21 µm in CT‐OS eyes (Figure 1F). After FDM induction, axial length increased progressively, reaching 3796 ± 29.93 µm in FDM‐OD and 3629 ± 14.85 µm in CT‐OS eyes at 16 weeks, corresponding to an interocular difference of 167.5 ± 28.28 µm (Figure 1F–H; Figure S1C, Supporting Information). Notably, the most rapid axial elongation occurred within the first 4 weeks post‐occlusion, with a difference of 119.0 ± 15.21 µm between eyes, while elongation during the following 8 and 12 weeks was slower but remained progressive.

Similar myopic development was observed in the LIM model: at 16 weeks, LIM‐OD eyes reached −2.83 ± 1.08 D, while CT‐OS eyes showed a hyperopic shift to 4.08 ± 1.07 D, resulting in a mean diopter shift of −6.92 ±1.63 D (Figure 1I,J). Axial length in LIM‐OD eyes increased to 3753 ± 21.25 µm, compared with 3664 ±15.20 µm in CT‐OS eyes, yielding an interocular difference of 102.3 ± 9.22 µm (Figure 1K–M; Figure S1D, Supporting Information). As with the FDM model, LIM mice also exhibited the fastest axial elongation during the first 4 weeks (105.0 ±17.66 µm); however, in contrast to the FDM model, axial growth plateaued thereafter, with minimal further elongation observed at 12 weeks post‐induction. These results confirm the successful establishment of a diabetic myopia mouse model using either FDM or LIM induction.

### 2.2 Mitigating DR Vascular Lesions Through Myopia Modeling

**Figure 2:**
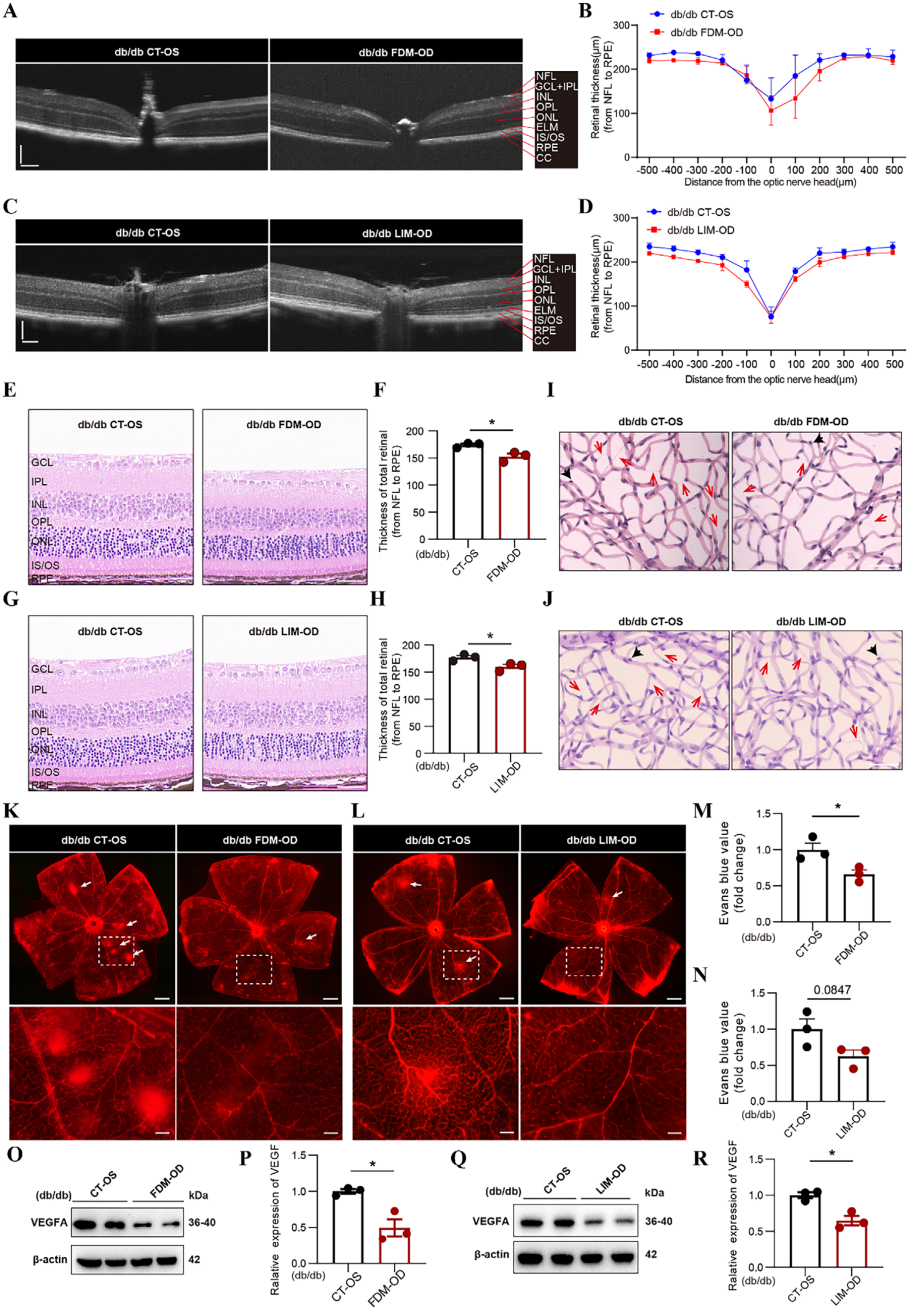
Myopia induced by FDM or LIM alleviates retinal vascular lesions and reduces VEGFA expression in db/db mice. A,B) Representative OCT images and retinal thickness statistics from the db/db CT‐OS and FDM‐OD eyes after moderation at 16 weeks. Retinal structural layers were annotated as follows: nerve fiber layer (NFL); ganglion cell layer (GCL); inner plexiform layer (IPL); inner nuclear layer (INL); outer plexiform layer (OPL); outer nuclear layer (ONL); external limiting membrane (ELM); inner segment/outer segment of photoreceptors (IS/OS); retinal pigment epithelium (RPE); choriocapillaris (CC); Scale bars indicate 100 µm. (*n* = 6/group). C,D) Representative OCT images and retinal thickness statistics from the db/db CT‐OS and LIM‐OD eyes after moderation at 16 weeks. Scale bars indicate 100 µm. (*n* = 6/group). E,F) Representative histopathological images (H&E staining) and quantification analysis of retinas (400 µm from optic nerve head) from the db/db CT‐OS and FDM‐OD retinas. Scale bars indicate 50 µm. (*n* = 3/group). G,H) Representative H&E staining and quantification analysis of retinas from the db/db CT‐OS and LIM‐OD retinas. Scale bars indicate 50 µm. (*n* = 3/group). I,J) Retinal trypsin digestion (PAS staining) of db/db CT‐OS and FDM‐OD retinas (I) or db/db CT‐OS and LIM‐OD retinas (J), with counts for acellular vessel numbers and endothelial/perivascular cell ratios. Acellular vessels are denoted by red arrowheads, and perivascular cells are indicated by black arrowheads. Scale bars indicate 50 µm. (*n* = 6/group). K,L) Representative retinal flatmount images of Evans Blue dye in db/db CT‐OS and FDM‐OD eyes (K) or db/db CT‐OS and LIM‐OD eyes (L). Sites of vascular leakage are denoted by white arrowheads. Scale bars indicate 500 µm; 100 µm for higher‐magnification images (boxed areas). (*n* = 3/group). M,N) Quantification of Evans blue extraction assays in db/db CT‐OS and FDM‐OD groups (M) or db/db CT‐OS and LIM‐OD groups (N). (*n* = 3/group). O,P) Western blot images illustrating the protein level of VEGFA in db/db CT‐OS and FDM‐OD groups. (*n* = 3/group). Q,R) Western blot images illustrating the protein level of VEGFA in db/db CT‐OS and LIM‐OD groups. (*n* = 3/group). Results expressed as mean ± SEM. ^*^ *p* <0.05. *P* values were determined by unpaired two‐tailed Student's *t* test (F,H,M,N,P,R).

To investigate the impact of myopia on DR, we conducted in vivo fluorescein sodium angiography (FFA) and utilized optical coherence tomography (OCT) to observe changes in retinal vasculature and thickness. Microaneurysms serve as the primary clinical marker of DR. On fundus photos and in FFA imaging, we observed tortuosity of the retinal vessels along with some leakage. The FFA results revealed numerous microaneurysms and occasional leakage points in the retinal vasculature of the db/db CT‐OS eyes (Figure S2A,B, Supporting Information). Notably, the number of microaneurysms significantly reduced in both db/db FDM‐OD and LIM‐OD eyes (Figure S2A,B, Supporting Information). OCT findings demonstrated a substantial thinning of both central and peripheral retinal thickness after establishing the myopia model, consistent with prior literature reports (**Figure** **2**A–D). Further validation was achieved through H&E staining of the retina 400 µm from the optic nerve. Similarly, both FDM‐OD and LIM‐OD eyes exhibited full‐thickness retinal thinning, particularly in the ONL, and the number of photoreceptor cells in the ONL was significantly decreased compared with the corresponding CT‐OS eyes (Figure 2E–H; Figure S2C,D, Supporting Information).

To delve deeper into whether myopia modeling could ameliorate microvascular lesions in DR, we procured eyeballs from these mice and isolated the retinas for Retinal trypsin digestion and periodic acid schiff (PAS) staining, Evans blue staining, and Western blot analysis of VEGFA expression. Retinal trypsin digestion flatmounts demonstrated a decrease in the number of avascular capillaries, an increase in perivascular cells, and a reduced endothelial/perivascular cell ratio in the db/db FDM‐OD eyes compared with the CT‐OS eyes (Figure 2I; Figure S2E,F, Supporting Information). In the LIM‐OD eye, a similar trend toward reduced acellular capillaries and increased perivascular support was observed compared to CT‐OS, although statistical significance was not reached (Figure 2J; Figure S2G,H, Supporting Information). Evans Blue staining depicted a mitigation in retinal leakage in the FDM‐OD eyes compared with the CT‐OS eyes (Figure 2K,M; Figure S2I, Supporting Information), and a comparable reduction in vascular leakage was also evident in the LIM‐OD eyes (Figure 2L,N; Figure S2J, Supporting Information). Additionally, Western blot analysis showed reduced VEGFA expression both in the retina of the FDM‐OD retina and LIM‐OD retina compared to the CT‐OS retina (Figure 2O–R). These findings suggest that myopia modeling may impede the progression of DR.

### 2.3 Enhanced Nna1 Expression in Müller Glia Induced by Myopia in Diabetic Mice

To investigate the intricate molecular and regulatory mechanisms underlying the inhibitory effects of myopia in inhibiting DR, we specifically selected db/db mice exhibiting elevated blood glucose levels exceeding 16.6 mmol l^−1^, significant myopic diopter shifting of −9D or more, and an axis elongation surpassing 100 µm for comprehensive single‐cell RNA sequencing (scRNA‐seq). As the FDM model had demonstrated robust and sustained axial elongation and a pronounced protective effect against DR in our initial experiments, we selected db/db FDM‐OD and control CT‐OS eyes for scRNA‐seq analysis.

**Figure 3:**
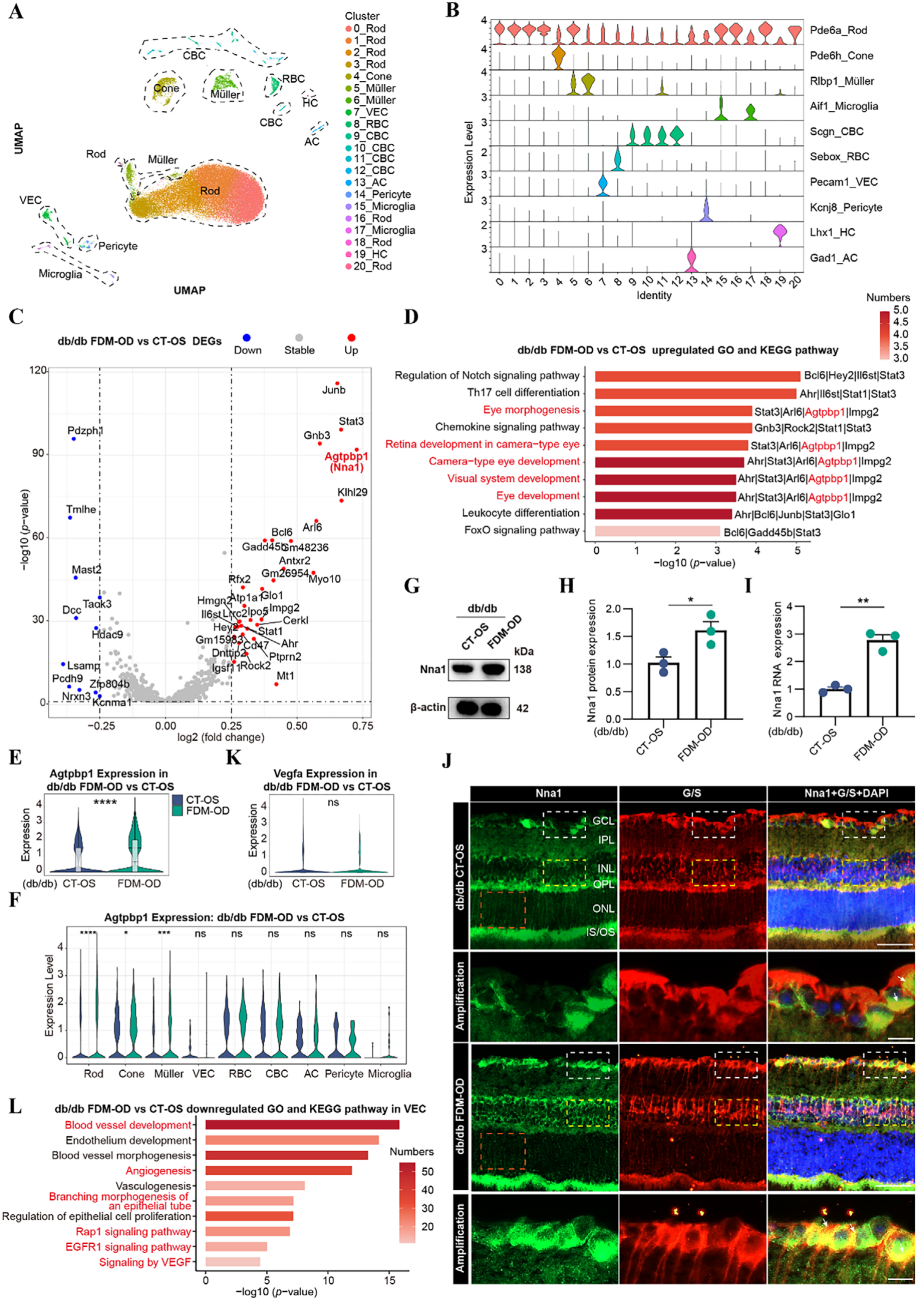
FDM modeling promoted the expression of Nna1 in db/db mice. A) Integrated UMAP clustering of retina cells from db/db FDM‐OD, db/db CT‐OS, db/db, and db/m mice. (*n* = 3/group). B) Violin plots of canonical markers for retina subsets from db/db FDM‐OD, db/db CT‐OS, db/db, and db/m groups. C) Volcano plot displaying upregulated (red dots) and downregulated (blue dots) DEGs in retina cells with the db/db FDM‐OD/CT‐OS comparison groups (|log2 fold change| > 0.25, *p* <0.05). D) Bar chart displaying representative GO terms and KEGG pathway enriched in upregulated DEGs of retina cells in the db/db FDM‐OD/CT‐OS comparison groups. Significance was determined using the cumulative hypergeometric distribution through the Metascape web tool. E,F) Violin plots of Agtpbp1 (Nna1) expression in all cells (E) and in retina subsets of db/db FDM‐OD and CT‐OS groups (F). G,H) Western blots images showing the protein level of Nna1 in db/db CT‐OS and FDM‐OD groups. (*n* = 3/group). I) Quantification of the expression of Nna1 in db/db CT‐OS and FDM‐OD groups measured by RT‐QPCR. (*n* = 3/group). J) Retina sections of db/db CT‐OS and FDM‐OD mice were processed for immunofluorescence using Anti‐Nna1 (Green) antibodies, G/S (Red), and DAPI (Blue). Scale bars indicate 50 µm; 10 µm for higher‐magnification images of the GCL layer (white boxed areas). Additional enlarged images of the INL layer (yellow boxed areas) and ONL layer (red boxed areas) are provided in Figure S4B and D. (*n* = 6/group). K) Violin plots of Vegfa expression in all cells of db/db FDM‐OD and CT‐OS groups. L) Bar chart displaying representative GO terms and KEGG pathway enriched in upregulated DEGs of VEC cells in the db/db FDM‐OD/CT‐OS comparison groups. Results expressed as mean ± SEM. ^*^ *p* <0.05, ^**^ *p* <0.01, ^***^ *p* <0.001, ^****^ *p* <0.0001. *P* values were determined by two‐tailed Wilcoxon rank‐sum test (E,F,K) or unpaired two‐tailed Student's *t* test (H,I).

Single‐cell suspensions derived from the retinas of db/db FDM‐OD, db/db CT‐OS, db/db, and db/m groups were meticulously processed into barcoded scRNA‐seq libraries for subsequent analyses. We identified 10 major retinal cell types, including rods (Rod) and cones (Cone) subgroups belong to photoreceptor cells, Müller and Microglia glial subgroups belong to glial cells, cone and rod bipolar subgroups belong to bipolar cells (CBC and RBC), vascular endothelial cells (VEC), pericyte cells (Pericyte), anaxonic cells (AC), and horizontal cells (HC) based on classical lineage markers (**Figure** **3**A,B; Figure S3A–C, Supporting Information). UMAP visualization demonstrated an even distribution of cells from all four groups across these major subclusters (Figure S3D, Supporting Information). However, due to the extremely low abundance of HC—fewer than three cells detected in the db/db group—they were excluded from subsequent analyses to ensure data robustness and statistical reliability (Figure 3A).

To identify the overall gene expression pattern, we first performed differentially expressed genes (DEGs) analysis between the db/db FDM‐OD and CT‐OS eyes. Compared with the CT‐OS eyes, the FDM‐OD eyes exhibited significantly upregulated expression genes involved in microtubule regulation and neuronal homeostasis (ATP/GTP binding protein 1 [Agtpbp1, also known as Nna1]) (TOP1),^[^ ^28^ ^]^ JAK/STAT signaling components (Stat3 and Stat1), AP‐1 transciption factor (JunB), protein degradation genes (Klhl29),^[^ ^29^ ^]^ cone‐specific genes (Gnb3),^[^ ^30^ ^]^ and more (Figure 3C). Conversely, downregulated genes in the db/db FDM‐OD group were linked to neural adhesion factors (Lsamp),^[^ ^31^ ^]^ microtubule‐associated factors (Mast2), and others (Figure 3C). Subsequent GO and KEGG analysis unveiled enriched pathways related to Notch signaling, Th17 cell differentiation, eye morphogenesis, visual and retina development, and the FoxO signaling in FDM‐OD eyes compared with CT‐OS eyes (Figure 3D). Particularly, the enrichment of visual development and eye morphogenesis pathways correlated with the upregulation of Nna1 (Figure 3D). Intriguingly, Nna1 has been shown to have a crucial function in the progression of CNS degeneration disease.^[^ ^32^ ^]^ It is also known as cytosolic carboxypeptidases 1 (CCP1) and function as a cytosolic carboxypeptidase involved in microtubule deglutamylation and neuronal maintenance.^[^ ^32^ ^]^ The significant upregulation of this molecule in db/db FDM‐OD group compared to CT‐OS group aroused our interest (Figure 3E). To determine whether this upregulation was cell‐type specific, we analyzed Nna1 expression across retinal cell subpopulations. Violin plots revealed marked elevation of Nna1 in Rod, Cone, and Müller glia in the db/db FDM‐OD group (Figure 3F). These findings were further validated by RT‐qPCR and Western blotting, which confirmed significant increases at both the mRNA and protein levels (Figure 3G–I). Additionally, immunofluorescence co‐staining with the Müller glial marker Glutamine Synthetase (G/S) demonstrated strong colocalization and elevated Nna1 expression in Müller cells across multiple retinal layers of FDM‐OD retinas, whereas its expression in photoreceptors nuclei within the ONL remained unchanged (Figure 3J; Figure S4A‐G, Supporting Information).

### 2.4 Suppression of Angiogenesis‐Related Pathways in VEC Following Diabetic FDM Induction

Given our earlier observation that VEGFA expression was modestly decreased in the db/db FDM‐OD group compared to the CT‐OS group, we investigated its expression pattern at the single‐cell level. However, while VEGFA showed a decreasing trend, the differences were not statistically significant in most subgroups (Figure 3K; Figure S5A, Supporting Information). GO and KEGG enrichment analyses between db/db FDM‐OD and CT‐OS retinas revealed that the upregulated pathways were predominantly enriched in Müller glia, including regulation of protein stability, neuron apoptotic process, cellular responses to stimuli, and so on (Figure S5B, Supporting Information). Conversely, downregulated pathways were concentrated in VEC, notably including the VEGF signaling pathway (Figure S5C, Supporting Information). Focused analysis of VEC showed a marked suppression of angiogenesis‐related pathways such as blood vessel development, angiogenesis, branching morphogenesis of an epithelial tube, and several as well as vascular signaling pathways, such as Rap1, EGFR, and VEGF (Figure 3L), suggesting that the observed VEGF downregulation may result from impaired endothelial signaling.

### 2.5 Downregulated Nna1 in Müller Glia of Diabetic Retinas

**Figure 4:**
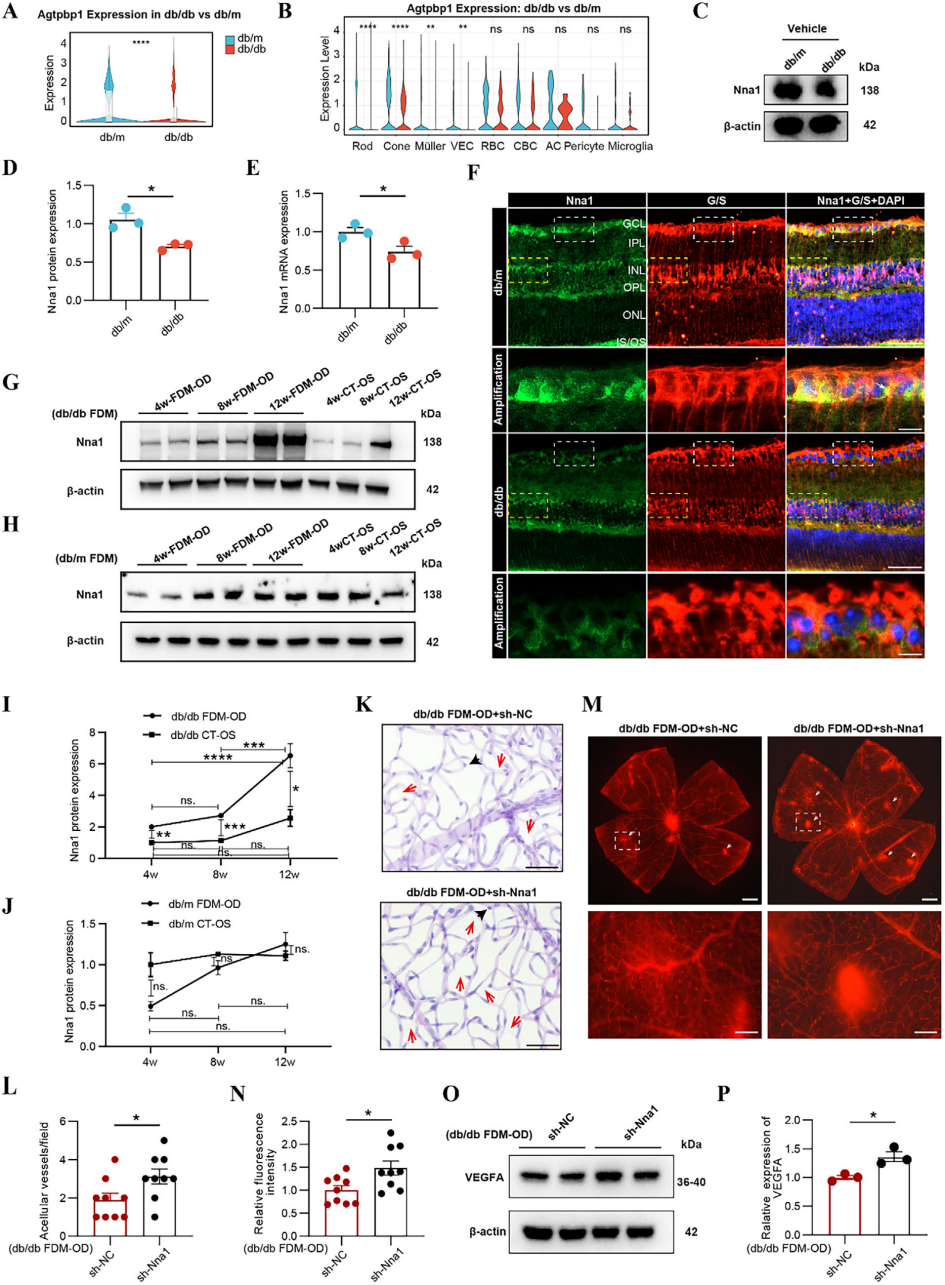
Downregulated Nna1 in db/db retina is involved in the protective effect of myopia against DR. A,B) Violin plots of Agtpbp1 (Nna1) expression in all cells (A) and in retina subsets of db/db and db/m groups (B). C,D) Western blots images showing the protein level of Nna1 in db/db and db/m groups. (*n* = 3/group). E) Quantification of the expression of Nna1 in db/db and db/m groups measured by RT‐QPCR. (*n* = 3/group). F) Retina sections of db/db and db/m mice were processed for immunofluorescence using Nna1 (green) antibodies, G/S (red), and DAPI (blue). Scale bars indicate 50 µm; 10 µm for higher‐magnification images of the GCL layer (white boxed areas). Additional enlarged images of the INL layer (yellow boxed areas) are provided in Figure S6B. G–J) Western blots images showing the protein level of Nna1 in 4w, 8w, 12w after db/db FDM model (G,I) or db/m FDM model (H,J), along with their corresponding CT‐OS eyes. (*n* = 3/group). K‐L) Retinal trypsin digestion (PAS staining) of FDM‐OD+sh‐NC and FDM‐OD+sh‐Nna1 group retinas, with counts for acellular vessel numbers. Scale bars indicate 50 µm. (*n* = 9/group). M,N) Representative retinal flatmount images of Evans Blue dye in FDM‐OD+sh‐NC and FDM‐OD+sh‐Nna1 eyes. Sites of vascular leakage are denoted by white arrowheads. Scale bars indicate 500 µm; 100 µm for higher‐magnification images (boxed areas). (*n* = 9/group). O,P) Western blot analysis of VEGFA protein expression in FDM‐OD eyes injected with sh‐Nna1 and sh‐NC. (*n* = 3/group). Results expressed as mean ± SEM. ^*^ *p* <0.05, ^**^ *p* <0.01, ^***^ *p* <0.001, ^****^ *p* <0.0001. *P* values were determined by two‐tailed Wilcoxon rank‐sum test (A, B), repeated Measures ANOVA (I,J), or unpaired two‐tailed Student's t test (D,E,L,N,P).

Our previous findings showed that Nna1 expression was significantly upregulated in the FDM‐OD eyes of diabetic mice, suggesting a potential role in the protective effect of myopia against DR progression. To further explore the baseline expression pattern of Nna1 in diabetic conditions, we compared 16‐week‐old db/db mice with age‐matched non‐diabetic db/m controls. Violin plot analysis revealed a significant reduction of Nna1 expression in the retinas of db/db mice (**Figure** **4**A). Subcluster analysis further demonstrated that this downregulation occurred across multiple retinal cell types, including Rod, Cone, Müller glia, and VEC (Figure 4B).

Western blot and qPCR results consistently confirmed the markedly reduced expression of Nna1 in db/db retinas compared to db/m controls (Figure 4C–E). Co‐localization of Nna1 with G/S revealed significantly reduced expression and colocalization of Nna1 in Müller glia across multiple retinal layers of db/db mice (Figure 4F; Figure S6A–D, Supporting Information). In contrast, double labeling with the vascular marker IB4 or the bipolar cell marker PKCα showed minimal overlap with Nna1, suggesting a cell‐type‐specific regulation (Figure S6E,F, Supporting Information). These results highlight that Nna1 is notably downregulated in Müller glia under diabetic conditions but upregulated in FDM‐treated eyes, implying that Nna1 may serve as a critical factor mediating the protective effects of myopia on DR.

### 2.6 Nna1 Knockdown Reverses the Protective Effect of Myopia on DR

To further validate whether the upregulation of Nna1 in db/db FDM‐OD eyes plays a critical role in mediating the protective effects against DR, we first investigated its dynamic expression patterns under different conditions. Our results showed Nna1 expression in the retinas of db/db mice progressively increased at 4, 8, and 12 weeks following FDM induction, with a significant elevation at 12 weeks post‐modeling (16 weeks of age) (Figure 4G,I). In contrast, no significant changes in Nna1 expression were detected in db/m mice subjected to the same FDM procedure (Figure 4H,J), suggesting that the upregulation of Nna1 may be specific to the co‐existence of myopia and diabetic conditions.

To determine whether Nna1 is functionally required for the FDM‐mediated protective phenotype, we intravitreally injected adeno‐associated viral (AAV) encoding Nna1‐targeting shRNA (sh‐Nna1) or a negative control vector (sh‐NC) into the right FDM‐OD eyes of 12‐week‐old db/db mice. We first assessed the transduction efficiency and localization of AAV‐shNna1. Since the AAV vector was engineered to express scarlet fluorescent protein, we examined retinal cryosections under a fluorescence microscope. The results showed robust expression of scarlet predominantly in Ganglion Cell Layer (GCL), Inner Plexiform Layer (IPL), Inner Nuclear Layer (INL), and Outer Plexiform Layer (OPL), confirming efficient viral infection in these regions (Figure S7A,B, Supporting Information). Then, Retinal trypsin digestion followed by PAS staining, Evans blue leakage assays, and Western blot analysis demonstrated that silencing Nna1 led to a significant increase in acellular capillaries, enhanced vascular leakage, and elevated VEGFA expression compared with the sh‐NC group (Figure 4K–P). These findings indicate that Nna1 is essential for mediating the protective effect of myopia on DR, and its knockdown exacerbates retinal vascular pathology.

### 2.7 Nna1 Overexpression Mitigates DR

**Figure 5:**
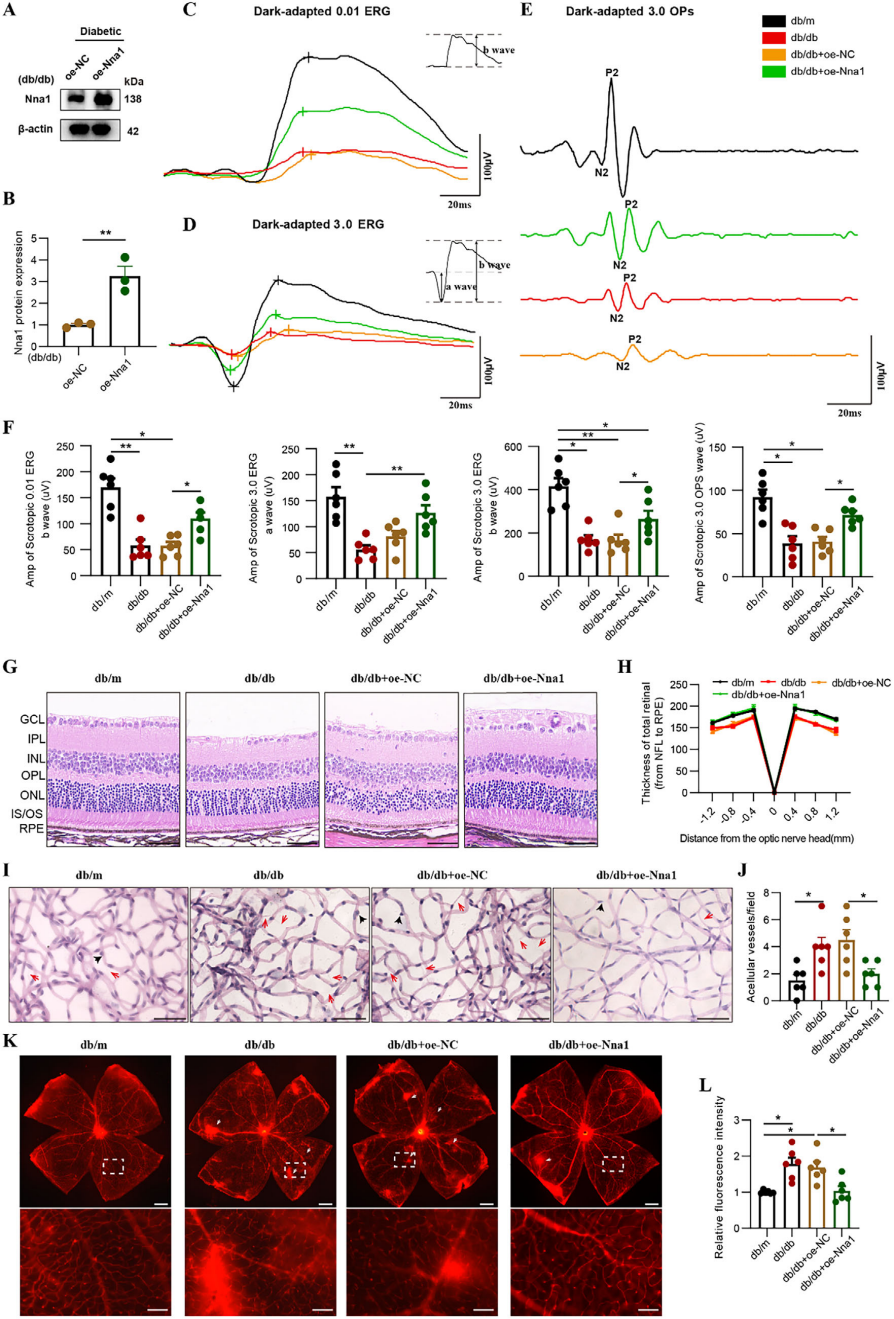
Nna1 overexpression alleviated DR. A,B) Western blots images showing Nna1 protein levels in oe‐NC and oe‐Nna1 groups of db/db mice. (*n* = 3/group). C,F) Scotopic 0.01 ERG and amplitude statistics (b‐wave) in db/m, db/db, db/db+oe‐NC, and db/db+oe‐Nna1 groups. (*n* = 6/group). D, F) Scotopic 3.0 ERG and amplitude statistics (a‐wave and b‐wave) in db/m, db/db, db/db+oe‐NC, and db/db+oe‐Nna1 groups. (*n* = 6/group). E,F) Scotopic 3.0 oscillatory potential ERG and OPs amplitude statistics in db/m, db/db, db/db+oe‐NC, and db/db+oe‐Nna1 groups. (*n* = 6/group). G,H) Representative H&E staining images and relative retina thickness statistics in db/m, db/db, db/db+oe‐NC, and db/db+oe‐Nna1 groups. Scale bars indicate 50 µm. (*n* = 3/group). I,J) Retinal trypsin digestion (PAS staining) of db/m, db/db, db/db+oe‐NC, db/db+oe‐Nna1 groups retinas, with counts for acellular vessel numbers. Acellular vessels are denoted by red arrowheads, and perivascular cells are pointed to by black arrowheads. Scale bars indicate 50 µm. (*n* = 6/group). K,L) Representative retinal flatmount images of Evans blue dye staining in db/m, db/db, db/db+oe‐NC, and db/db+oe‐Nna1 groups. Sites of vascular leakage are denoted by white arrowheads. Scale bars indicate 500 µm; 100 µm for higher‐magnification images (boxed areas). (*n* = 6/group). Results expressed as mean ± SEM. ^*^ *p* <0.05, ^**^ *p* <0.01. *P* values were determined by one‐way ANOVA (F, J, L).

To further confirm the function of Nna1 in DR, we generated lentiviral vectors to overexpress mouse gene Nna1 (oe‐Nna1) or the negative control (oe‐NC). RT‐qPCR showed significantly higher Nna1 mRNA levels in the overexpression group (db/db+oe‐Nna1) compared with the control vector group (db/db+oe‐NC) (Figure S8A, Supporting Information). Additionally, Western blotting demonstrated increased Nna1 protein expression in the overexpression group compared with the control group (**Figure** **5**A,B). Immunofluorescence staining further demonstrated that intravitreal injection of the oe‐Nna1 lentivirus markedly enhanced Nna1 expression in multiple retinal layers, particularly in the GCL, IPL, INL, and OPL (Figure S8B,C, Supporting Information). Notably, baseline Nna1 expression was significantly reduced in db/db and db/db+oe‐NC retinas compared to db/m controls, whereas Nna1 expression was robustly restored in the db/db+oe‐Nna1 group (Figure S8B,C, Supporting Information).

We then investigated Nna1's impact on retinal function in diabetic conditions using electroretinogram (ERG) tests. In the scotopic 0.01 ERG, the b‐wave amplitude reflects the retinal response from rods. We observed a decrease in the b‐wave amplitude of the db/db group compared with the db/m group, while treatment with oe‐Nna1 alleviated this mitigation compared with the oe‐NC group (Figure 5C,F). Scotopic 3.0 ERG, reflecting the combined rod‐cone response, showed a reduction in the a‐wave amplitude of the db/db group compared to the db/m group (Figure 5D,F). Conversely, the a‐wave amplitude increased in the db/db+oe‐Nna1 group compared to the db/db+oe‐NC group. The db/db and db/db+oe‐NC groups showed decreased b‐wave amplitudes, while a significant recovery was noted within the db/db+oe‐Nna1 group (Figure 5D,F). Oscillatory potentials (OPs) representing inner retinal function declined in the db/db group compared with the db/m group, while oe‐Nna1 treatment improved the OPs amplitude compared with the db/db+oe‐NC group (Figure 5E,F).

Next, we conducted H&E staining of retinas from the db/m, db/db, db/db+oe‐NC, and db/db+oe‐Nna1 groups. Our findings revealed thinner retinal layers in the db/db and db/db+oe‐NC groups compared with the db/m group (Figure 5G,H). Treatment with oe‐Nna1 notably increased retinal thickness (Figure 5G,H). Moreover, Retinal trypsin digestion flatmounts demonstrated an elevation in the count of avascular capillaries and a decrease in perivascular cells in the db/db group compared with the db/m group (Figure 5I,J). Administering with Nna1 overexpressed virus can invert this change (Figure 5I,J). Consistently, Evans blue leakage assays demonstrated increased vascular leakage in db/db mice, which was markedly attenuated by Nna1 overexpression (Figure 5K,L). These results suggest that Nna1 overexpression can protect the neuron and vascular structure and function of the diabetic retina.

### 2.8 Overexpressed Nna1 Attenuates Autophagy and Apoptosis Activation in db/db Mice

**Figure 6:**
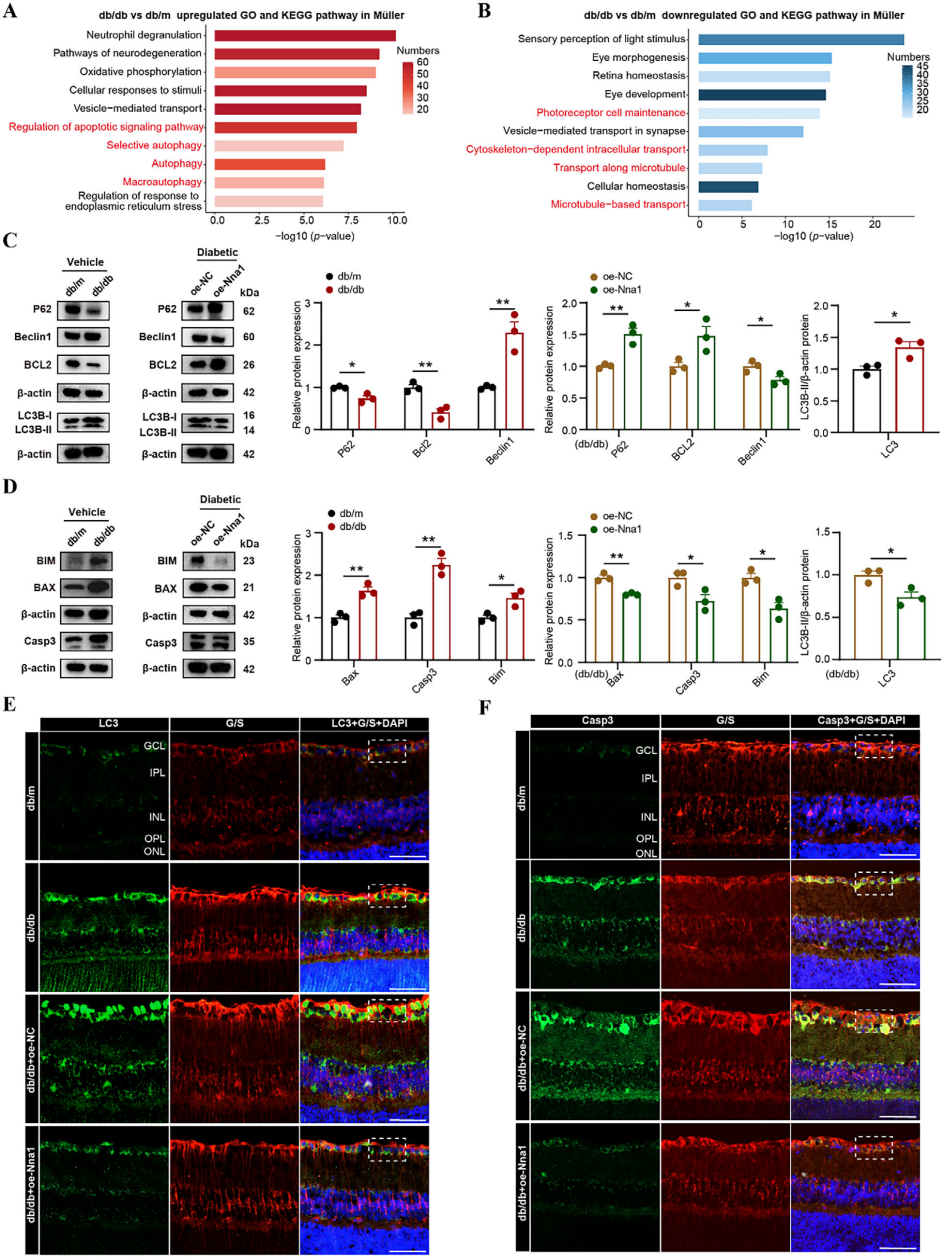
Nna1 overexpression alleviated DR through inhibited autophagy and apoptosis pathway. A,B) Bar charts displaying representative enriched GO terms and KEGG pathways in upregulated DEGs (A) and downregulated DEGs (B) of Müller cells between db/db and db/m groups. C) Western blots images depicting the protein levels of P62, Bcl2, Beclin1, and LC3B in db/m, db/db, or db/db+oe‐NC and db/db+oe‐Nna1 groups. (*n* = 3/group). D) Western blots images displaying the protein levels of Casp3, Bax, and Bim in db/m, db/db, or db/db+oe‐NC and db/db+oe‐Nna1 groups. (*n* = 3/group). E,F) Immunofluorescence images of retina sections from db/m, db/db, db/db+oe‐NC, and db/db+oe‐Nna1 mice, stained with LC3 (green), G/S (red) (E) or Casp3 (green), G/S (red) (F), and DAPI (blue). Enlarged images (white boxed areas) are provided in Figure S9C‐D. Scale bars indicate 50 µm. (*n* = 3/group). Results expressed as mean ± SEM. ^*^ *p* <0.05, ^**^ *p* <0.01. Statistical significance was assessed with an unpaired two‐tailed Student's t test (C,D).

Our previous findings identified Nna1 as a key mediator of the protective effect of myopia against DR, and demonstrated that Nna1 overexpression significantly mitigates DR progression. Given that Nna1 expression was primarily altered in Müller cells, we conducted further analysis specifically within the Müller cell subpopulations. In the Müller subgroup of db/db mice, gene set enrichment analysis revealed upregulation of pathways related to neurodegeneration, oxidative phosphorylation, apoptotic signaling, and autophagy, while pathways linked to retina homeostasis, microtubule organization, and intracellular transport were downregulated (**Figure** **6**A,B). Notably, previous studies have reported that Nna1 knockout (*pcd*) mice exhibit altered autophagy and apoptosis in cerebellar Purkinje cells,^[^ ^33^, ^34^, ^35^ ^]^ prompting us to hypothesize that Nna1 might be privotal in DR by modulating apoptotic and autophagy pathways.

To investigate this hypothesis, we examined canonical markers of autophagy and apoptosis. Microtubule‐associated protein light chain 3 (LC3) is a gold standard for measuring autophagy, while Beclin1 is a well‐established marker of autophagy in mammalian cells.^[^ ^36^, ^37^ ^]^ Our results revealed an increase in the LC3B‐II and Beclin1 protein levels in the db/db retinas compared with the db/m retinas, with a decrease observed in the db/db+oe‐Nna1 retinas compared with the db/db+oe‐NC retinas (Figure 6C). P62/SQSTM1 is an adaptor protein that links ubiquitinated cargo to autophagosomes for degradation, and its reduced levels are commonly interpreted as a marker of enhanced autophagic flux.^[^ ^38^ ^]^ B‐cell lymphoma 2 (Bcl‐2) is an important regulatory protein that can inhibit autophagy by forming a complex with Beclin‐1, and it also inhibits apoptosis in cells.^[^ ^39^ ^]^ Our findings showed reduced P62 and Bcl‐2 in the db/db retinas compared to db/m retinas (Figure 6C). Following intravitreal application of oe‐Nna1, P62 and Bcl‐2 increased, while Beclin1 decreased compared to the oe‐NC retinas (Figure 6C). BCL2‐like protein 11 (Bim), BCL2‐associated X protein (Bax), and Caspase‐3 are key regulators and markers of apoptosis.^[^ ^40^ ^]^ We observed an increase in Bax, Bim, and Caspase‐3 in the db/db group, which decreased after Nna1 overexpression, indicating that apoptosis is upregulated in db/db mice but can be suppressed by Nna1 (Figure 6D). Immunofluorescence results further validated the upregulation of LC3 and Caspase‐3 in Müller cells of db/db mice, whereas Nna1 overexpression markedly suppressed their expression (Figure 6E,F; Figure S9A–E, Supporting Information). These findings indicate that both autophagy and apoptosis are activated in Müller cells under diabetic conditions, and Nna1 overexpression can effectively inhibit these pathological processes in db/db mice.

### 2.9 Nna1 Modulates Autophagy and Apoptosis In Vitro by Regulating Tubulin Polyglutamylation

**Figure 7:**
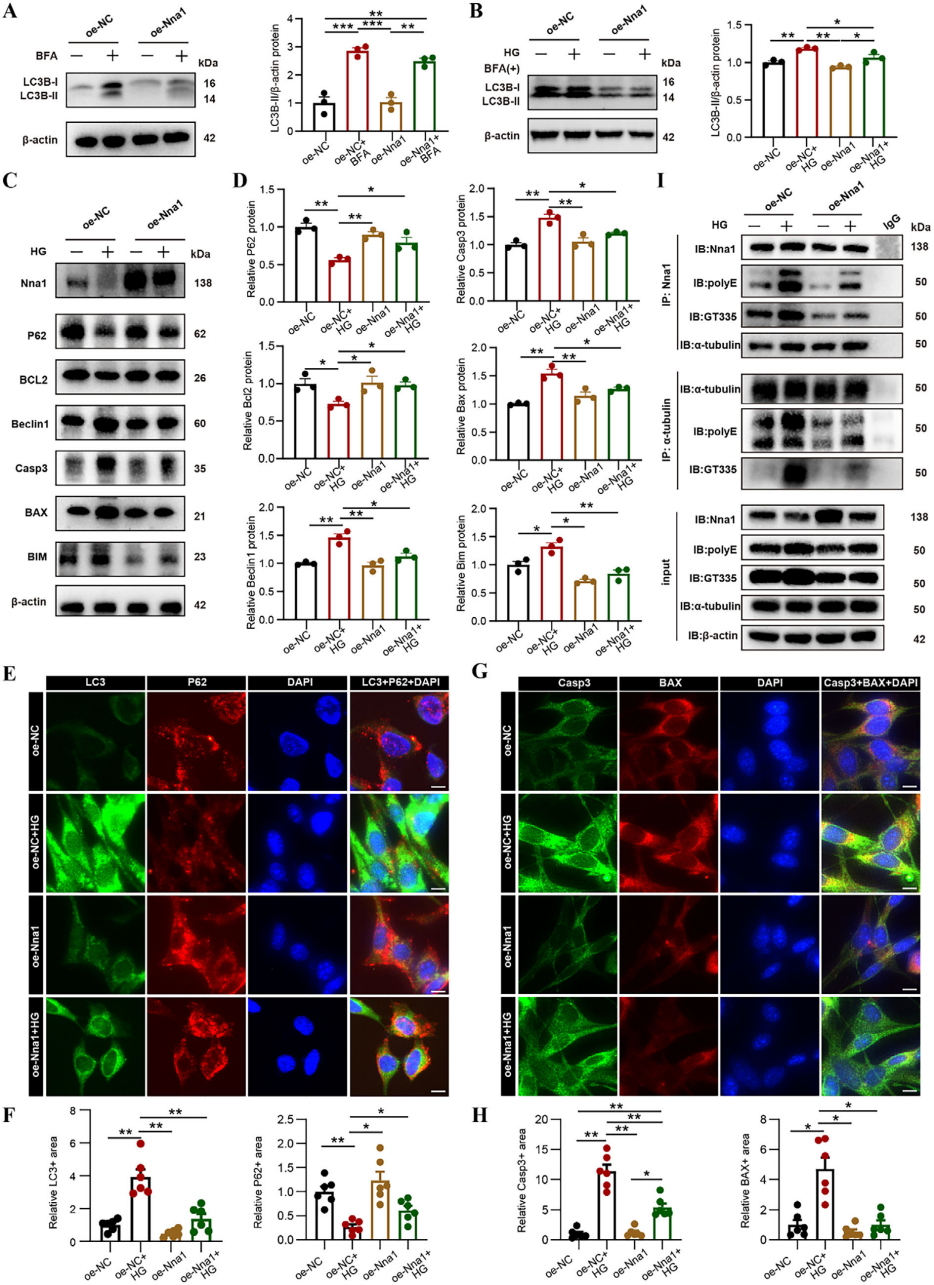
Nna1 overexpression inhibited HG‐induced autophagy and apoptosis pathways in rMC‐1 cells by regulating Tubulin Polyglutamylation. A‐B) Western blots images depicting the protein expression of LC3B in rMC‐1 cells transfected with oe‐NC or oe‐Nna1. BFA (400 nm) was administered to the groups for 6 h prior to protein extraction. (*n* = 3/group). C,D) Western blots images showing the protein level of Nna1, P62, Bcl2, Beclin1, Casp3, Bax, and Bim in rMC‐1 cells treated with HG or untreated, and transfected with oe‐NC or oe‐Nna1. (*n* = 3/group). E,F) Immunofluorescence staining and quantifications of rMC‐1 cells transfected with oe‐NC or oe‐Nna1 with or without HG treatment. Cells were stained with LC3 (green), P62 (red), and DAPI (Blue). Scale bars indicate 10 µm. (*n* = 6/group). G,H) Immunofluorescence staining and quantifications of rMC‐1 cells transfected with oe‐NC or oe‐Nna1 with or without HG treatment. Cells were stained with Casp3 (green), BAX (red), and DAPI (Blue). Scale bars indicate 10 µm. (*n* = 6/group). I) The binding affinity between Nna1 and a‐tubulin and its effect on polyglutamylation tubulin after Nna1 overexpression with or without HG treatment was evaluated using Co‐IP assays. (*n* = 3/group). Results expressed as mean ± SEM. ^*^ *p* <0.05, ^**^ *p* <0.01, ^***^ *p* <0.001. *P* values were determined by one‐way ANOVA (A,B,D,F,H).

rMC‐1 cells act as an in vitro model of retina Müller cells, whereas 661 W cells serve as an in vitro model of retinal photoreceptors. Both rMC‐1 and 661 W cells were exposed to high glucose conditions (HG, 50 mmol l^−1^). Notably, HG treatment did not significantly alter Nna1 expression in 661 W cells, whereas it led to a marked reduction of Nna1 in rMC‐1 cells (Figure 7C; Figure S10A–E, Supporting Information). Therefore, rMC‐1 cells were chosen for subsequent in vitro studies. Furthermore, Nna1 levels was significantly restored after transfection with an Nna1 overexpression lentivirus (Figure 7C; Figure S10C–E, Supporting Information). To assess autophagic flux in rMC‐1 cells under Nna1 overexpression, bafilomycin A1 (BFA) was used to block autophagic flux. As presented in **Figure** **7**A, LC3B‐II protein levels significantly increased following BFA treatment, indicating enhanced autophagic flux. We then examined changes in LC3B‐II protein levels under HG‐treatment in both control and Nna1 overexpression groups. The results revealed that LC3B‐II protein expressions were markedly reduced in the Nna1 overexpression group compared with the control group (Figure 7B). In line with this, the autophagy‐related protein Beclin1 was upregulated, while the levels of P62 and Bcl‐2 were decreased in HG‐treated cells, suggesting increased autophagic activity (Figure 7C,D). Nna1 overexpression reversed these effects, leading to increased expression of P62 and Bcl‐2, and decreased levels of Beclin1 and LC3B‐II (Figure 7C,D). Furthermore, the expression of apoptosis markers, including Bim, Bax, and Caspase‐3, was significantly elevated in the HG group, while Nna1 overexpression effectively suppressed their expression (Figure 7C,D). Immunofluorescence results revealed that LC3, Caspase‐3, and BAX expression were upregulated and P62 expression was downregulated under HG stimulation, whereas Nna1 overexpression reversed these changes, resulting in reduced LC3, Caspase‐3 and BAX, and elevated P62 levels (Figure 7E–H). These findings indicate that Nna1 overexpression inhibits HG‐induced autophagy and apoptosis.

**Figure 8:**
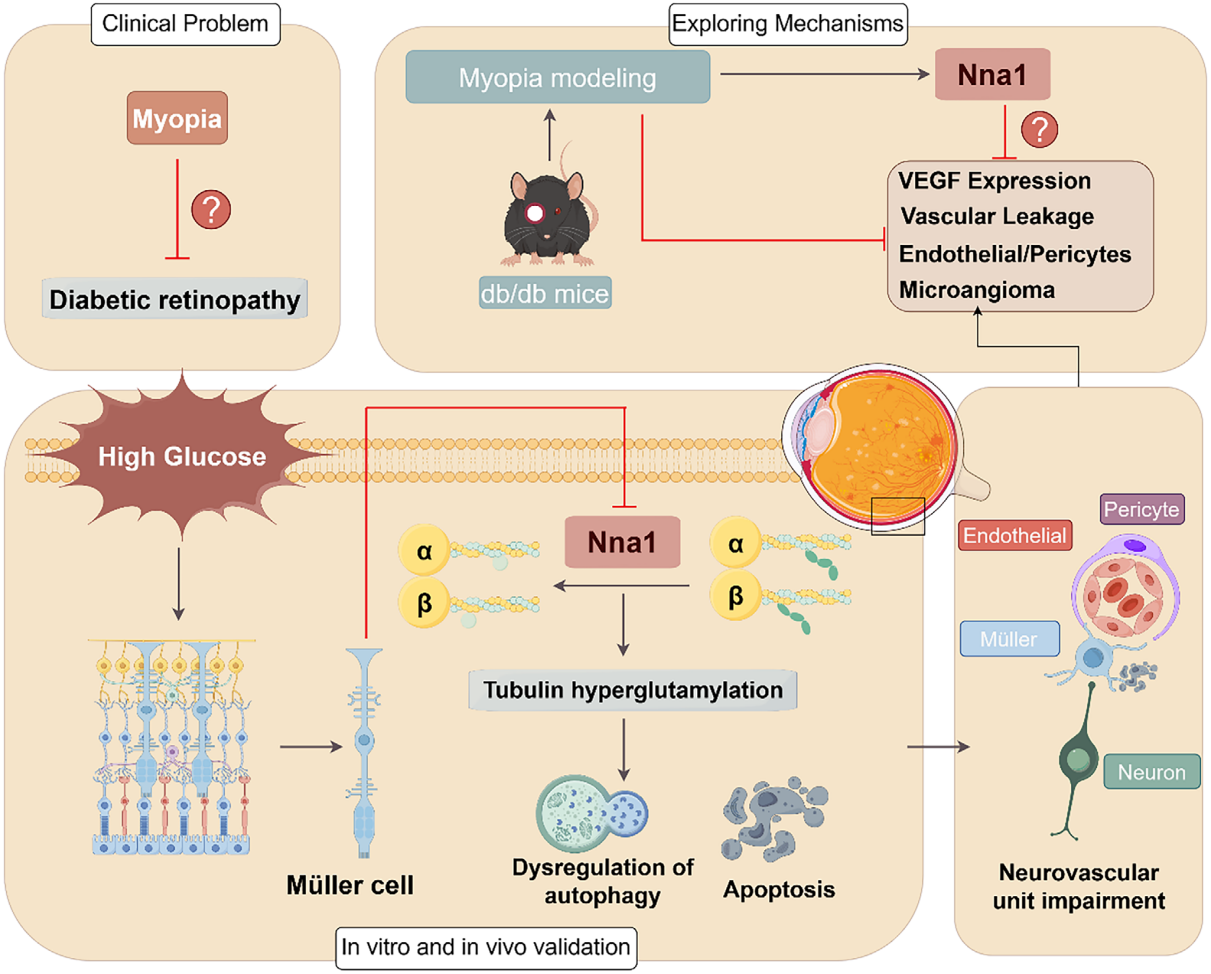
Schematic overview of the mechanistic pathways investigated in this study. The relationship between myopia and DR has long been of clinical interest. In diabetic mice, Nna1 expression is downregulated, whereas in diabetic mice with FDM, Nna1 expression is upregulated–particularly in Müller cells–accompanied by decreased VEGF expression, reduced vascular leakage, a lower endothelial‐to‐pericyte ratio, and fewer microaneurysms. Overexpression of Nna1 was found to inhibit diabetic retinal neurovascular unit damage by regulating autophagy and apoptosis in Müller cells through the inhibition of tubulin hyper‐glutamylation. (By Figdraw).

Nna1, as a deglutamylase of tubulin, plays a crucial role in regulating microtubule dynamics. In mice with Nna1 mutations, there's a notable manifestation of neuron apoptosis due to the hyperglutamylation of microtubule proteins.^[^ ^34^ ^]^ Building upon this understanding, we hypothesized that Nna1 might modulate the level of tubulin hyperglutamylation in DR. To explore this, we first performed molecular docking using ZDOCK. The top‐ranked model revealed that Tyr1138 on Nna1 is in close contact with Lys336 on α‐tubulin, with an interaction distance of ≈3.2 Å, indicating a likely binding interface (Figure S11A,B, Supporting Information). Notably, Lys336 is located near the C‐terminal region of α‐tubulin, which is enriched in polyglutamylation sites. This spatial proximity supports a direct regulatory mechanism whereby Nna1, modulates tubulin polyglutamylation levels via physical interaction with α‐tubulin. This binding was further confirmed by co‐immunoprecipitation assays (Figure 7I). Furthermore, in rMC‐1 cells cultured under high‐glucose conditions, we observed a significant increase in polyglutamylated tubulin (PolyE) and GT335 (specific for polyglutamylated tubulin) levels in both Nna1‐ and α‐tubulin‐immunoprecipitated complexes as well as in whole‐cell lysates, which was markedly reduced upon Nna1 overexpression (Figure 7I; Figure S12A, Supporting Information). PolyE and GT335 serve as indicators of tubulin polyglutamylation.^[^ ^41^ ^]^ PolyE generally detects longer glutamate side chains, while GT335 specifically recognizes the branching point of glutamate side chains on tubulin.^[^ ^41^ ^]^ Immunofluorescence analysis revealed that both GT335 or PolyE colocalized with LC3, and HG treatment enhanced their expression, whereas Nna1 overexpression suppressed the colocalization of GT335 and PolyE with LC3 (Figure S12B–D, Supporting Information), suggesting that GT335 and PolyE are directly associated with autophagosomes and may facilitate microtubule‐dependent autophagosome transport. These results indicate that Nna1 directly interacts with α‐tubulin to regulate tubulin polyglutamylation levels. Under diabetic (HG) conditions, increased tubulin polyglutamylation induces activation of autophagy and apoptosis pathways, contributing to cellular dysfunction. Overexpression of Nna1 reduces tubulin polyglutamylation, thereby attenuating excessive autophagy and apoptosis and protecting retinal cells from damage (**Figure** **8**).

## 3 Discussion

Epidemiological and clinical studies have reported an inverse association between high myopia and the severity or progression of DR.^[^ ^7^, ^10^, ^11^, ^12^, ^13^, ^14^, ^15^, ^16^, ^17^, ^23^ ^]^ Several large‐scale cohort studies, including those in Asian populations, have shown that individuals with high myopia tend to exhibit a lower prevalence and milder grades of DR.^[^ ^7^, ^14^, ^15^, ^16^ ^]^ This protective association is thought to result from axial elongation in myopic eyes, which leads to retinal thinning, reduced retinal capillary pressure, and lower metabolic demand.^[^ ^19^, ^20^, ^23^ ^]^ These anatomical and physiological alterations may collectively contribute to reduced retinal hypoxia, lower VEGF expression, and diminished vascular leakage. However, these proposed mechanisms remain largely speculative and lack direct experimental validation, particularly in animal models.

In this study, we investigated the interplay between myopia and DR, aiming to elucidate the protective mechanisms conferred by myopia against DR. We utilized spontaneous type II diabetic db/db mice and established myopia models using both FDM and LIM approaches by covering the right eye. Through comprehensive measurements—including measurements of axial length, refractive error, blood glucose levels, and fundus imaging—we successfully generated diabetic mouse models with myopic phenotypes. Histological (H&E staining) and structural (OCT) analyses revealed a generalized retinal thinning in db/db mice following either FDM or LIM induction, affecting both central and peripheral regions. This observation is consistent with previous studies reporting axial elongation‐induced retinal thinning in myopic eyes.^[^ ^42^, ^43^ ^]^ Interestingly, DR has also been associated with retinal thinning, particularly in advanced stages.^[^ ^44^ ^]^ In our model, the thinning was more pronounced in the outer retinal layers, especially the photoreceptor layer. This could represent a potential mechanism by which myopia confers protection against DR. Photoreceptors are among the most metabolically active cells in the retina, with the highest oxygen consumption. In the context of myopia‐induced photoreceptor reduction—coupled with hyperglycemia‐induced neurodegeneration in DR—the decreased photoreceptor density likely reduces overall retinal oxygen demand.^[^ ^20^ ^]^ This diminished oxygen consumption may alleviate hypoxia‐induced VEGF expression, thereby mitigating retinal neovascularization and leakage. Notably, this mechanism is reminiscent of panretinal photocoagulation therapy in DR, which reduces retinal oxygen consumption by ablating peripheral photoreceptors and thereby preserve macular function.^[^ ^24^ ^]^

Moreover, consistent with prior studies, our findings confirmed the protective role of myopia in DR, particularly in suppressing retina vascular lesions. Specifically, both FDM and LIM modeling induced a significant elongation of the axial length, and led to a reduction in the number of microaneurysms and acellular capillaries, decreased retinal vascular leakage, and lower VEGF expression levels in DR. Interestingly, this is in contrast to the well‐documented VEGF upregulation observed in both DR and myopic pathologies such as choroidal neovascularization (CNV), where hypoxia is a key driving force.^[^ ^45^, ^46^ ^]^ The paradoxical downregulation of VEGF in myopic eyes with DR suggests that although both conditions independently promote VEGF expression, their underlying anatomical and pathophysiological contexts differ. In DR, retinal capillary dropout and pericyte loss induce inner retinal hypoxia, especially affecting the neurovascular unit, which stimulates VEGF‐driven neovascularization and vascular leakage.^[^ ^46^ ^]^ Conversely, high myopia is characterized by choroidal thinning and reduced choroidal perfusion, leading to choroidal ischemia, particularly at the RPE–Bruch's membrane–choriocapillaris complex.^[^ ^45^ ^]^ This contributes to RPE atrophy, lacquer cracks, and focal VEGF upregulation that promotes CNV originating from the choroid.^[^ ^45^ ^]^ Since the choroid provides the primary blood supply to the outer retina, reduced perfusion can result in degeneration and thinning of the outer retinal layers.^[^ ^27^ ^]^ The consequent decrease in photoreceptor density may lower retinal oxygen consumption, thereby alleviating hypoxia‐induced VEGF expression, the key drivers of DR progression.

scRNA‐seq has emerged as a powerful tool in deciphering molecular mechanisms at the single‐cell level.^[^ ^47^, ^48^ ^]^ To unravel the underlying mechanisms contributing to the protective effect of myopia against DR, we conducted scRNA‐seq on whole retina tissues from db/db eyes with or without FDM induction. Our data unveiled a spectrum of DEGs associated with inflammation, signaling cascades, and neurodegeneration protection in the diabetic with myopia mice group. Pathway analyses further underscored the activation of inflammatory responses and immune signaling in this group, consistent with previous findings.^[^ ^49^, ^50^ ^]^ Consistent with our previous findings that myopia attenuates vascular abnormalities in DR, VECs from the diabetic with myopia group exhibited significant downregulation of angiogenesis‐related pathways. These included vasculature development, endothelial cell proliferation and migration, and blood vessel morphogenesis, as well as key signaling pathways such as VEGF, EGFR and RAP1. This repression of pro‐angiogenic signaling may contribute to the observed attenuation of vascular pathology in diabetic retinas with myopic modifications.

Notably, Nna1, a tubulin deglutamylase implicated in neuroprotection, was identified as the top upregulated gene in diabetic Müller glia under myopic conditions. Originally identified by Adalynn Harris et al. in the spinal motor neuron cell bodies of mice with sciatic nerve compression injury, Nna1 contains a nuclear localization signal and ATP/GTP binding sites, suggesting roles in neuronal differentiation and regeneration.^[^ ^51^ ^]^ Subsequent studies have demonstrated that Nna1 is a critical player in neurodegenerative processes, exerts key functions in maintaining neuronal integrity and function.^[^ ^52^ ^]^ These functions encompass the regulation of microtubule dynamics, facilitation of protein degradation pathways, and modulation of autophagic processes.^[^ ^53^ ^]^ Dysregulation of Nna1 has been closely associated with neurodegenerative conditions.^[^ ^54^ ^]^ Nna1 knockout mice exhibit features of *Purkinje cell degeneration*, commonly known as *pcd* mice.^[^ ^32^ ^]^ Recent research indicates that *pcd* mice manifest degenerative changes not only in Purkinje neurons but also in neurons of the auditory thalamic,^[^ ^55^ ^]^ mitral valve neurons in the olfactory bulb,^[^ ^56^ ^]^ and photoreceptor neurons in the retina,^[^ ^57^, ^58^ ^]^ albeit with varying degrees. Our findings revealed a specific upregulation of Nna1 in Müller cells of db/db mice with FDM‐induced myopia, whereas its expression was downregulated in Müller cells of db/db mice without myopia. Notably, the upregulation of Nna1 was associated with signaling pathways related to eye morphogenesis and visual system development, suggesting its potential involvement in retinal structural and functional regulation. To test its role, we performed intravitreal injection of AAV‐shRNA to knock down Nna1 expression. This intervention weakened the protective effect of myopia on DR and increased VEGFA expression, indicating that Nna1 is a key mediator of the myopia‐conferred protection against retinal vascular pathology. Conversely, overexpression of Nna1 in the retina of db/db mice preserved both vascular and neural integrity and suppressed VEGFA expression, further confirming the critical protective role of Nna1 in DR.

Müller cells are the principal macroglia in the retina and the only cells that span the entire retinal thickness.^[^ ^59^ ^]^ They are intimately involved in maintaining the structure and function of the neurovascular unit, which includes retinal neurons, endothelial cells, and pericytes.^[^ ^59^ ^]^ Dysfunction of Müller cells disrupts this unit, contributing to capillary leakage, endothelial proliferation, and neuronal degeneration.^[^ ^59^ ^]^ Therefore, the downregulation of Nna1 in db/db Müller cells may represent a pathogenic factor in DR, while its upregulation under myopic conditions likely plays a protective role against DR progression. To further explore the downstream mechanisms by which Nna1 protects against DR, we analyzed pathway alterations in Müller cells. Notably, apoptosis and autophagy‐related signaling pathways were significantly activated in the Müller cell subgroup. Macroautophagy/Autophagy, also known as “self‐digestion,” is a cellular process crucial for metabolizing and degrading intracellular substances, including damaged organelles, misfolded proteins, and large molecules, by forming autophagosomes. These autophagosomes fuse with lysosomes to create autolysosomes, where the substrates undergo degradation by lysosomal enzymes.^[^ ^60^ ^]^ Autophagy is vital for normal development and response to environmental stresses in organisms, playing a significant role in maintaining proper retinal function.^[^ ^60^ ^]^ Previous researches have demonstrated a connection between Nna1 and the autophagy process.^[^ ^53^ ^]^ Research on Nna1 mutant *(pcd)* mice has shown increased levels of autophagy in various neurons, including cerebellar Purkinje cells, hypothalamus cells, cortex, and amygdala cells before the manifestation of neuron degeneration.^[^ ^61^ ^]^ Similarly, electron microscopy observations have revealed an abundance of autophagy‐like vesicles in the retinal photoreceptor cell layer of *pcd* mice.^[^ ^58^ ^]^ Moreover, studies have linked abnormal levels of autophagy to the occurrence of DR, contributing to damage in VEC, VEGF release, neovascularization, and even neuroepithelial layer damage in the retina.^[^ ^62^ ^]^ Devi et al. reported that HG condition induces mitophagy, a form of macroautophagy that especially targets damaged mitochondria.^[^ ^63^ ^]^ Kong et al. also demonstrated enhanced autophagy in diabetic mice.^[^ ^64^ ^]^ However, it's important to note that autophagy in DR can have dual effects – supporting cell survival under mild stress while triggering programmed cell death under severe conditions.^[^ ^65^ ^]^ The autophagy process involves essential components such as the Atg12‐Atg5‐Atg16L and LC3 complexes, which are crucial for phagophore elongation and autophagosome closure.^[^ ^66^ ^]^ Beclin‐1, in combination with Vps34, is crucial in facilitating autophagosome formation, lipid membrane extension, autophagosome maturation, and cargo accumulation.^[^ ^67^ ^]^ P62 acts as a cargo receptor, attaching cellular cargo to the autophagosome membrane and facilitating its degradation by lysosomes. Lowe levels of P62 levels typically indicate increased autophagy activity.^[^ ^38^ ^]^ In our research, we observed an elevation in LC3B‐II and Beclin‐1 expression, along with a reduction in P62 and Bcl‐2 levels in db/db mice, indicative of activated autophagy in db/db mice. Apoptosis, a process of programmed cell destruction, involves important regulators such as the Bcl‐2 family, including the anti‐apoptotic Bcl‐2 gene and the pro‐apoptotic Bax gene.^[^ ^68^ ^]^ Additionally, the caspase family, especially Caspase‐3, is critical in activating the apoptotic pathway.^[^ ^69^ ^]^ Our findings showed upregulation of Bax, Caspase‐3, and Bim expression in db/db mice, suggesting increased apoptosis in this context. To further investigate into the function of Nna1 in DR, we use Nna1 overexpression virus and introduced it into db/db mouse retinas or transfected Müller rMC‐1 cells. Remarkably, we observed that overexpression of Nna1 inhibited the activation of both autophagy and apoptosis in the retinas of DR mice and in HG‐treated rMC‐1 cells. This protective effect translated into preserved Müller cell function, reduced retinal cell apoptosis, and increased retinal thickness. These findings highlight the potential therapeutic value of Nna1 in mitigating DR‐related cellular damage.

Tubulins, which include free dimeric α‐ and β‐tubulins as well as microtubules, are ubiquitous in nucleated organisms. They play critical roles in maintaining normal cell structure, cell migration, division, and various physiological functions. Glutamylation is one of the most common genetic and enzymatic types of posttranslational modification of tubulin proteins.^[^ ^70^, ^71^, ^72^ ^]^ Cells influence microtubule function by regulating the balance of tubulin glutamylation/deglutamylation.^[^ ^73^ ^]^ Nna1 is a glutamylase known for its role in modulating tubulin glutamylation. Studies have shown that Nna1 knockout in pcd mice leads to the accumulation of highly glutamylated microtubule proteins, resulting in endoplasmic reticulum stress and cell apoptosis.^[^ ^33^, ^34^ ^]^ Consistent with these reports, our findings provide direct evidence that Nna1 interacts with tubulin to regulate polyglutamylation levels under diabetic conditions. We observed a marked accumulation of hyperglutamylated tubulins in both Nna1‐ and α‐tubulin–immunoprecipitated complexes, as well as in whole‐cell lysates following HG treatment. Notably, Nna1 overexpression attenuated this accumulation in rMC‐1 cells, further supporting its regulatory role. Moreover, our immunofluorescence analysis revealed that hyperglutamylated tubulins are directly associated with autophagosomes and may facilitate microtubule‐dependent autophagosome transport.

The specific upregulation of Nna1 exclusively in the co‐existing disease model points to a pathophysiological synergy between myopia and diabetes. We propose the following sequence of events: First, the mechanical stretch from axial elongation in myopia induces Müller cells to elongate their processes, creating a structurally adapted and potentially vulnerable retina.^[^ ^74^, ^75^, ^76^, ^77^ ^]^ Then, upon the onset of diabetes, this pre‐adapted tissue is exposed to metabolic stress from advanced glycation end products, which disrupt cytoskeletal homeostasis—partly through tubulin hyperglutamylation potentially exacerbated by suppressed Nna1.^[^ ^70^, ^72^, ^78^, ^79^ ^]^ We hypothesize that the prior structural remodeling in myopia “primes” Müller cells, rendering them more sensitive to the secondary metabolic insult and triggering a compensatory Nna1 upregulation. Given the established roles of Nna1 in regulating microtubule stability, autophagy, and neuronal survival,^[^ ^32^, ^33^, ^34^ ^]^ its induction is thus positioned as a critical adaptive response to preserve retinal integrity under combined stress.

In summary, our study uncovers a previously unrecognized mechanism by which myopia confers protection against DR. To our knowledge, this is the first study to establish diabetic mouse models combined with two distinct myopia models and experimentally validate the longstanding clinical observation that highly myopic patients rarely develop DR. We demonstrate that myopia modeling suppresses microvascular lesions and VEGFA expression in diabetic mice. More importantly, through scRNA‐seq, we identify Nna1 as a Müller cell‐specific protective factor whose expression is remarkably upregulated under the coexistence of diabetes and myopia. Mechanistically, Nna1 acts as a tubulin deglutamylase, protecting Müller cells by reducing tubulin polyglutamylation and thereby preventing excessive activation of autophagy and apoptosis signaling. This preservation of Müller cell integrity helps maintain the neurovascular unit, safeguards photoreceptor survival, and reduces VEGFA‐driven vascular abnormalities. Notably, even though myopia‐induced retinal stretching still leads to photoreceptor loss, the concurrent reduction in VEGFA and photoreceptor density may collectively decrease retinal hypoxia and vascular leakage, thus mitigating DR progression. These findings provide a mechanistic explanation for the epidemiological phenomenon linking myopia with reduced DR risk and highlight Nna1 as a potential therapeutic target. While our study advances understanding of DR pathogenesis, limitations remain, including the incomplete delineation of Nna1‐mediated signaling cascades and the lack of human data. Future research integrating patient‐derived retinal samples will be crucial for clinical translation and may open a new avenue for preventing or treating DR by targeting Nna1.

## 4 Experimental Section

### Animals

Male db/db mice (type II diabetic, C57BL/KsJ‐Lepr^db/db^) and db/m controls were utilized in this study, and raised in controlled conditions of temperature and lighting. Specific pathogen‐free facilities were utilized for housing the animals. Blood glucose levels and body weights were monitored monthly, with mice exhibiting fasting blood glucose levels surpassing 16.9 mmol l^−1^ classified as diabetic. All animal experimental procedures were evaluated and authorized by the Animal Care and Use Committee at the Laboratory Animal Research Center, Xiangya Medical School, Central South University (No. 202409159).

### Establishment of FDM and LIM Models

Light‐proof test tubes were meticulously fashioned into uniform eye patches measuring ≈8 mm in diameter with a peripheral edge of ≈1 mm, specifically designed for visual deprivation in db/db mice. After administering a 1% solution of pentobarbital sodium via intraperitoneal injection for anesthesia, the eye patch was expertly sutured around the right eye of each mouse using 4–0 sutures. The positioning of the eye patch's edge was carefully set far enough from the eyelids to avoid functional interference. Moreover, a plastic collar was attached around the neck of each mouse to prevent accidental displacement of the eye patch. Regular checks were conducted three times daily to identify any abnormalities such as unusual secretions, signs of infection, or eye patch detachment, ensuring uninterrupted visual deprivation of the mice's experimental eyes. Data from mice experiencing frequent occluder loss or exhibiting eye diseases were omitted from the analysis to ensure the integrity of the experimental outcomes.

To induce LIM, a −10 D concave lens with a diameter of ≈10–12 mm was mounted into a lightweight cylindrical plastic frame. The lens assembly was created by attaching the lens to the opening of a balloon nozzle using glue, forming a stable and custom‐fit structure suitable for the mouse eye. The assembled lens unit was then positioned in front of the right eye of each mouse and fixed using elastic bands or sutures to ensure optical alignment and secure placement throughout the experiment. Before lens installation, one drop of levofloxacin eye drops was applied to the right eye to prevent infection. Mice were monitored daily for signs of lens displacement, eye discharge, or inflammation. Animals showing frequent lens loss or ocular abnormalities were excluded from subsequent analysis. The untreated left eye served as a control.

### Axis Length Assessments

The ocular axial length was assessed with a Global Swept‐Source OCT device (YG‐100K MAX, TowardPi Medical Technology Ltd, Beijing). Each measurement involved capturing at least five traces per eye, which were subsequently analyzed offline. In this study, we present exclusively the optical axial lengths, determined by adding the anterior chamber depth, lens thickness, vitreous chamber depth, and retina thickness.

Briefly, mice were anesthetized, and their pupils were dilated using compound tropicamide eye drops (5 mg mL^−1^). One operator positioned the mouse on a mouse holder and gently opened its eyelids to fully expose the eyeball. Meanwhile, another operator adjusted the focus and position of the OCT device to capture the largest cross‐section of the eyeball. The distance from the cornea to the outer surface of the retina was then measured, providing the axial length of the eye.

### Refraction Assessments

Refraction measurements were conducted in darkness by two experienced optometrists using the traditional slit‐lamp retinal examination method. Prior to measurements, mice received 0.5% compound tropicamide eye drops in both eyes. Anesthesia was not administered during refraction. In a darkened room, the experimenter gently held the mice to expose the eye being examined, while the optometrist, positioned at a 50 cm working distance, used handheld lenses with 0.5D intervals to perform retinoscopy along both horizontal and vertical meridians. Astigmatism was calculated using half equivalent spherical lenses. Measurements were conducted three times, recorded in diopters, and the average refractive power of each eye of each mouse was recorded.

### Fluorescein Fundus Angiography

Retinal vascular permeability in mice was assessed using fluorescein angiography. The mice were put under anesthesia, and their eye pupils were fully dilated. Subsequently, the eyeball was coated with carbomer to keep the cornea moist. Fundus examinations were conducted 5 min after intraperitoneal injection of 0.1 mL of 5% fluorescein sodium using the Phoenix Image‐guided Micron IV system (Phoenix Technology Group, CA, USA).

### Optical Coherence Tomography

The retinal thicknesses were measured using a retinal imaging system (Phoenix Image‐guided Micron IV OCT system, CA, USA). Following adequate anesthesia and dilate the pupils, mice were positioned on a bracket. The bracket was adjusted to bring the mouse's eye gradually closer to the OCT lens, while continuously adjusting the machine's angle and focus to center the mouse's optic disc in the field of view. After adjusting the contrast, gamma value, and brightness, images were captured and retinal thicknesses were measured.

### Electroretinography

Mice were subjected to at least 12 h of darkness prior to being anesthetized with sodium pentobarbital. After adequate anesthesia and dilate the pupils, the mice were placed on a heating pad (37 °C). Two active electrodes were placed on the corneal surface, two reference electrode was attached on the cheek skins, and a ground electrode was clipped to the skin on the back. Electroretinography was performed using an ocular electrophysiological detector (RETI Port/Scan 21; Roland, Germany) with full‐field stimulation. The stimuli consisted of scotopic flashes at intensities of 1.0 and 3.0 cd.s m^−^^2^, as well as OPs at 3.0 cd.s m^−^^2^, which were recorded.

### Evans Blue Assay

Mice were tail vein injected with a 2% Evans blue solution in normal saline. Following a 2 h period, mice were euthanized, and perfusion with PBS was conducted to remove Evans blue dye from blood vessels. Retinas were meticulously dissected, dried, and weighed. Subsequently, the retinas were placed in formamide at 70 °C overnight to remove the Evans blue dye. The extract was then subjected to centrifugation, and the absorbance of the supernatant was quantified at 620 nm utilizing a spectrophotometer to evaluate blood‐retinal barrier integrity. Furthermore, retinal flat mounts were also prepared after perfusion, and examined under a fluorescence microscope to visualize Evans blue fluorescence and assess the extent of retinal leakage.

### Intravitreal Injection

To manipulate Nna1 expression in vivo, both overexpression and knockdown strategies were applied using lentiviral and adeno‐associated viral vectors, respectively. Lentivirus overexpressing the mouse Nna1 gene and the negative control vector was generated and purified by OBIO Technology (Shanghai, China). When the db/db mice reached 12 weeks of age, they were anesthetized, and their pupils were dilated. Subsequently, employing a 33‐gauge needle and a surgical microscope, either the oe‐Nna1 or oe‐NC vector was intravitreally administered (2 µL per eye). After a 4‐week period, ocular samples were collected for various analytical assessments.

For gene knockdown, an AAV2/2 vector encoding Nna1‐targeting shRNA and a negative control vector, both carrying a red fluorescent reporter (mScarlet), were also obtained from OBIO Technology. Intravitreal injections were similarly performed with 2 µL of viral suspension per eye using the same procedure. Mice were sacrificed 4 weeks after injection for molecular and histological evaluations.

### Molecular Docking and Visualization

The amino acid sequences of Nna1 (UniProt ID: Q641K1) and α‐tubulin (UniProt ID: P68373) were retrieved from the UniProt database. The predicted PDB files were submitted to the ZDOCK protein–protein docking server (http://zdock.umassmed.edu/), with α‐tubulin set as the ligand and Nna1 as the receptor. Default parameters were used for the docking procedure. The top‐scoring docking complex was selected based on ZDOCK score ranking. Visualization and analysis of the docking model were performed using PyMOL (version 4.6, Schrödinger LLC), with protein structures shown in cartoon representation. The binding interface between Nna1 and α‐tubulin was further highlighted in magnified views for clarity.

### Cell Culture and Treatment

The rat retina Müller rMC‐1 cell line and mouse photoreceptor‐derived 661 W cell line, provided by Aolu Biotechnology (Shanghai, China), were cultured under standard conditions of 5% CO_2_ at 37 °C and maintained in DMEM supplemented with 10% fetal bovine serum (Gibco) and 1% penicillin/streptomycin. To establish in vitro DR cell models, 50 mm D‐glucose (HG group) was added to the cell culture medium and administered for 48 h. Another group of rMC‐1 cells administered with 25 mm glucose used as the negative control group. To induce autophagy, cells were treated with 100 nm BFA (S1413, Selleck). Following a 6 h incubation, further experiments were performed.

### Establishment of a Stable Cell Line with Nna1 Overexpression

Nna1 overexpressing or control vector lentivirus was transfected rMC‐1 to establish stable cell line. In brief, cells were seeded at 1 × 10^5^ per well in a six‐well plate cultured overnight. Lentivirus (5 × 10^6^ TU mL^−1^) and polybrene (5 µg mL^−1^) were added for infection. After 24 h, culture medium was replaced, and cells were cultured for an additional 48 h with puromycin (2 µg mL^−1^), yielding a stable Nna1‐overexpressing rMC‐1 cell line. RNA and protein were extracted to verify a stable overexpressed Nna1 cell line.

### Western Blot

Mice retinas and rMC‐1 cells were obtained and lysed in a lysis buffer supplemented with a protease inhibitor cocktail and a phosphatase inhibitor. Then, the samples were sonicated for 10 s and set on ice for 30 min. After centrifugation at 4 °C to collect the supernatant, protein concentration was detected with a BCA assay Kit (Epizyme, China). After boiling at 100 °C for 10 min, the proteins were loaded on SDS–PAGE gels and transferred to PVDF membranes. Then, the membrane was blocked with 5% skim milk at room temperature for 2 h, followed by overnight incubation with the primary antibody at 4 °C. Next, the membrane was incubated with the secondary antibody for 1 h at room temperature. Protein gray intensity was quantified using ImageJ software (1.8.0). Details of the primary antibodies can be found in Table S3 (Supporting Information).

### RT‐qPCR

RNA was extracted from retinal tissues and rMC‐1 cells with Cell/Tissue RNA purification Kit (ES Science, Dalian, China). After measuring the RNA concentration, reverse transcription to cDNA was performed following the manufacturer's instructions. For quantitative real‐time PCR, SYBR Green Premix (Takara, Dalian, China) was utilized as per the manufacturer's protocol. Primers were designed and obtained from Sangon Biotechnology Company Ltd. β‐actin served as the internal reference. Relative gene expression was analyzed using the 2^−ΔΔCt^ method. The primer sequence for Nna1 can be found in Table S2 (Supporting Information).

### Co‐Immunoprecipitation

Cultured rMC‐1 cells were harvested after different treatments and were lysed in ice‐cold IP lysis buffer supplemented with protease and phosphatase inhibitors (Beyotime, China). The lysates were incubated on ice for 30 min and then centrifuged at 12000 ×g for 15 min at 4 °C to remove cell debris. The supernatants were collected and quantified using a BCA protein assay kit (Thermo Scientific).

Immunoprecipitation was performed using the Protein A/G Magnetic Beads IP Kit (Abbkine, KTD104) according to the manufacturer's instructions. Briefly, 1 µg of antibody against Nna1 (Proteintech), α‐tubulin(Proteintech), GT335 (AdipoGen), or PolyE (AdipoGen) — or corresponding control IgG—was incubated with 20 µL of Protein A/G magnetic beads at room temperature for 30 min. After washing, 1 mg of total cell lysate was incubated with the antibody‐bound beads overnight at 4 °C with gentle rotation.

The beads were then thoroughly washed with lysis buffer to remove non‐specific binding. Immunocomplexes were eluted by boiling the beads in 1× SDS loading buffer at 95 °C for 5 min, and subsequently analyzed by SDS‐PAGE and western blotting using the indicated antibodies.

### Hematoxylin and Eosin Staining

Mice eyes were separated and immersed in FAS ocular fixative solution (Servicebio, China) overnight. After experiencing dehydration and paraffin immersion, paraffin sections (4 µm) were prepared and stained with hematoxylin‐eosin staining solution following standard protocols. The retinal structure and thickness were then observed using a light microscope.

### Retina Trypsin Digestion and Periodic Acid Schiff Staining

Mice retinas underwent trypsin digestion following a previously described method. Briefly, mice eyes were separated and fixed in 4% paraformaldehyde (PFA) for 48 h. Then, mice retinas were dissected and washed by PBS overnight before submitting to digestion in 6% trypsin 1:250 (Biofroxx; USA) for 60 min at 37 °C. Then, the retinal vascular network was isolating and stained with PAS. A light microscope was utilized to examine acellular vessels, endothelial cells, and pericytes.

### Immunofluorescence Staining

Mouse eyes were dissected and immersed in 4% PFA overnight at 4 °C. After removing the 4% PFA, the eyes were immersed in 20% sucrose for 8 h, then in 30% sucrose overnight for gradient dehydration. Following that, the eyeballs were embedded in O.C.T compound (SAKURA Tissue Tek, West Chester, PA). Retinal sections (10 µm) were cut and blocked with 5% BSA and 0.3% Triton X‐100 for 60 min at room temperature.

For cell immunofluorescence, rMC‐1 cells were seeded onto glass coverslips and subjected to the indicated treatments. The coverslips were then fixed with ice‐cold methanol for 15 min, followed by blocking in 5% BSA for 60 min at room temperature.

After blocking, both tissue sections and cell coverslips were incubated with primary antibodies overnight at 4 °C and incubated with appropriate secondary antibodies for 60 min the next day at room temperature. Fluorescence images were observed using an immunofluorescence slice scan (Pannoramic DESK, Hungary) or confocal laser scanning microscopy and measured using ImageJ software.

### scRNA‐Seq Data Preparation

The Chromium Next GEM Single Cell 3ʹ Reagent Kit (10×Genomics, 1000268) version 3.1 was used to prepare the scRNA‐seq libraries. Sequencing was applied to the Illumina NovaSeq 6000 PE150 System. The sequencing data were processed utilizing the CellRanger software pipeline (version 7.0.1). For data integration and clustering, the Seurat package (version 4.3.0) in R (version 4.2.2) was applied. Cells of low quality were removed, specifically those with fewer than 200 detected genes or a mitochondrial gene percentage greater than 40%.

Cell counts were transformed using the ‘NormalizeData’ function to achieve log‐normalization. Dimensionality reduction was carried out with the ‘RunPCA’ function. To visualize the cells, a 2D UMAP algorithm was applied via the ‘RunUMAP’ function. Clusters of interest were identified using the ‘FindNeighbors’ and ‘FindClusters’ functions. Marker genes for each cluster were pinpointed with the ‘FindAllMarkers’ function.

### Differentially Expressed Genes Analysis

DEGs were identified using the FindMarker function in Seurat software. Significant differential expression was determined based on the criteria of a with a *p* value <0.05 and |log2fold change| >0.25.

### GO Analysis

The web‐based platform Metascape^[^ ^80^ ^]^ (www.metascape.org) was utilized to perform GO and pathway analyses based on the input of DEGs. Among the top 50 enriched GO terms encompassing a range of cell types, 10 GO terms or pathways related to diseases were identified. The ggplot2 package (version 3.4.1) was applied to visualize these results.

### Statistical Analysis

All data were expressed as mean ± SEM and analyzed with two‐tailed Student's t test, one‐way ANOVA, or Repeated Measures ANOVA using GraphPad Prism 8.0 (GraphPad Software, San Diego, CA, USA). *p* value of 0.05 or less was recognized as statistically significant.

## Conflict of Interest

The authors declare no conflict of interest.

## Author Contributions

L.X. and Y.C. are co‐first authors and contributed equally to this work.  L.X. and Y.C. performed the experiments and wrote the manuscript. B.C. and B.L. collected and analyzed data. J.Z. and H.X. conceived experiments, supervised, and revised the project. All authors discussed and approved the manuscript.

## Supporting information



Supporting Information

Supplemental Table 1

Supporting Information

## Data Availability

The data that support the findings of this study are available from the corresponding author upon reasonable request.
