# Supplementary material for: Single‐Cell RNA Sequencing of Retina Reveals Nna1 Upregulation in Myopic Diabetic Retinopathy as a Protective Factor Against Diabetic Damage
Source: Adv Sci (Weinh). 2025 Nov 5;13(5):e00438. doi: 10.1002/advs.202500438 (PMC12849878; doi:10.1002/advs.202500438)
Supplement: Supplementary file 1 — Supporting Information [file ADVS-13-e00438-s003.docx]

**Supplementary Information**

**Single-cell RNA sequencing of retina reveals Nna1 upregulation in myopic diabetic retinopathy as a protective factor against diabetic damage**

1. **Supplementary Figures:**

**Supplementary Figure 1** Establishment of a combined myopia and diabetic mouse model using FDM and LIM approaches.

**Supplementary Figure 2** Myopia induced by FDM or LIM alleviates retinal pathology in db/db mice.

**Supplementary Figure 3** Marker-based classification of retinal cell populations in scRNA-seq data.

**Supplementary Figure 4** Colocalization analysis of Nna1 with Müller glia in db/db FDM-OD and CT-OS groups.

**Supplementary Figure 5** Differential gene expression and pathway changes across retinal cell types in db/db FDM-OD compared to CT-OS eyes based on scRNA-seq.

**Supplementary Figure 6** Colocalization analysis of Nna1 with Müller glia in db/db and db/m groups and Double immunofluorescence staining of Nna1 with vascular and bipolar cell markers.

**Supplementary Figure 7** Validation of shNna1-AAV transduction efficiency.

**Supplementary Figure 8** Immunofluorescence staining of retinal Nna1 expression following lentiviral-mediated overexpression.

**Supplementary Figure 9** Enlarged views and the negative results of immunofluorescence images shown in Figure 6E–F.

**Supplementary Figure 10** High glucose reduces Nna1 expression in rMC-1 cells, which is reversed by overexpression.

**Supplementary Figure 11** Predicted molecular docking model between Nna1 and α-tubulin.

**Supplementary Figure 12** LC3 colocalization with GT335 and PolyE in rMC-1 cells.

1. **Supplementary Tables (refer to the corresponding supplementary file):**

**Supplementary Table 1** Marker genes used for identification of major retinal cell types in scRNA-seq.

**Supplementary Table 2** Primer sequences used in this study.

**Supplementary Table 3** Primary antibodies used in this study.

**Supplementary Table 4** Up and Downregulated DEGs of Total cells db/db FDM-OD vs. db/db CT-OS.

**Supplementary Table 5** Up and Downregulated DEGs of Total cells db/db vs. db/m.

**Supplementary Table 6 A-I** Up and Downregulated DEGs of subcluster cells db/db FDM-OD vs. db/db CT-OS.

**Supplementary Table 7 A-I** Up and Downregulated DEGs of subcluster cells db/db vs. db/m.

**
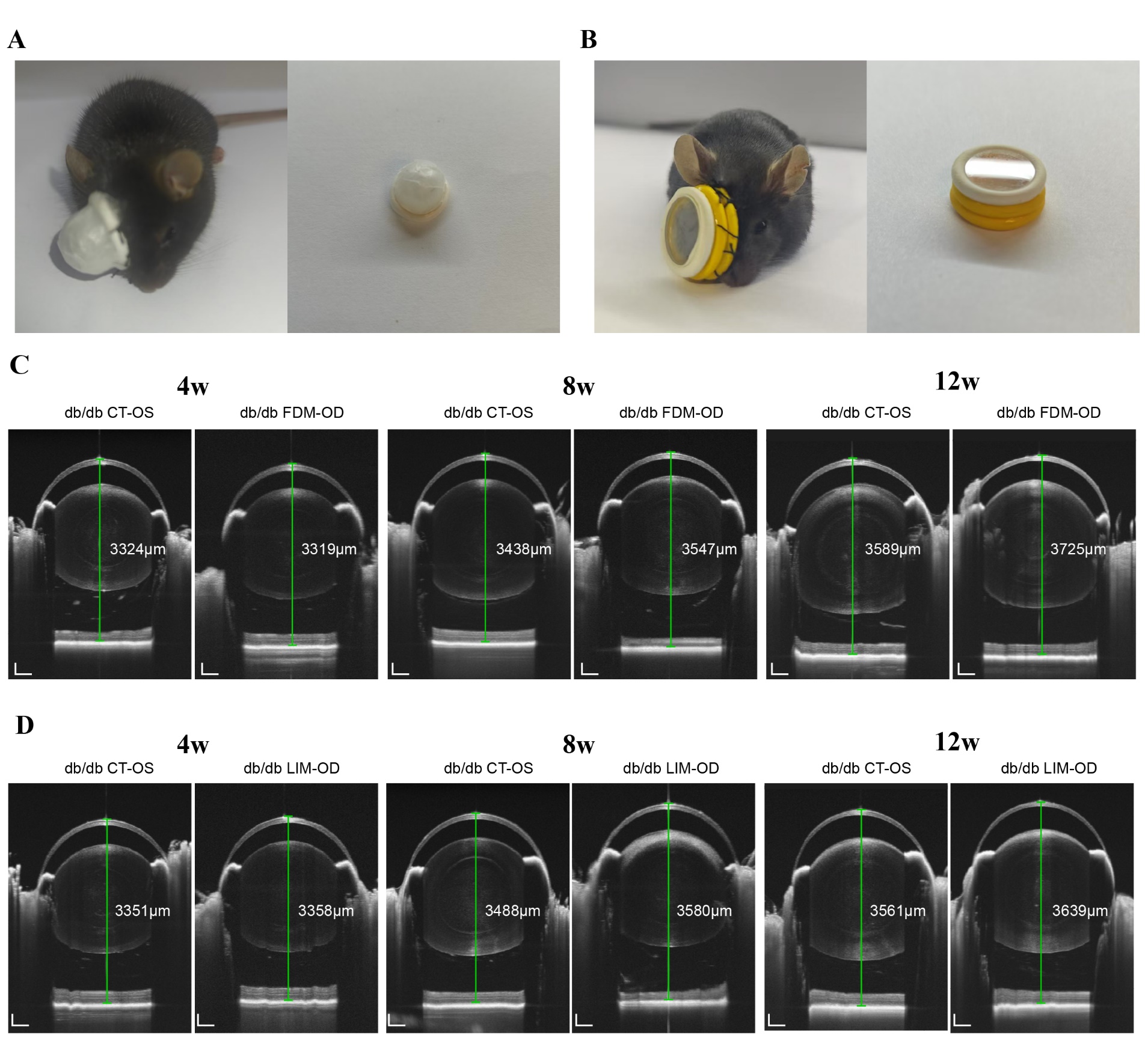
**

**Supplementary Figure 1 Establishment of a combined myopia and diabetic mouse model using FDM and LIM approaches. (A-B)** Representative images of myopia models in 4 weeks db/db mice. FDM model established by covering the right eye with a translucent diffuser (A). LIM model established by mounting a −10D concave lens over the right eye (B). **(C-D)** Representative images of axial length measurements obtained by global-swept-source OCT at 4, 8, and 12 weeks in db/db CT-OS and FDM-OD eyes (C), and in CT-OS and LIM-OD eyes (D). Scale bars indicate 200 μm.

**
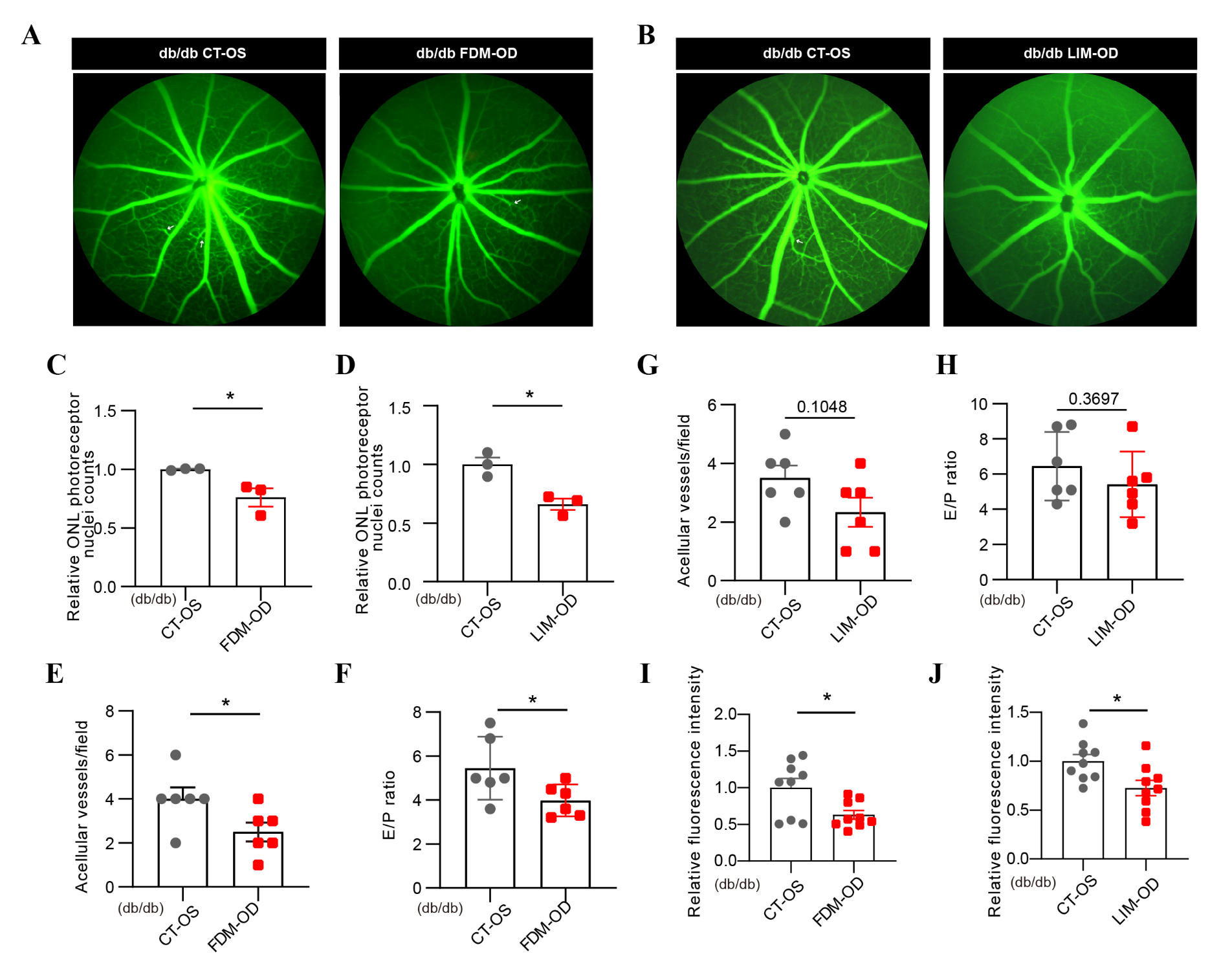
**

**Supplementary Figure 2 Myopia induced by FDM or LIM alleviates retinal pathology in db/db mice. (A-B)** Representative FFA images depicting the CT-OS and FDM-OD fundus (A) or CT-OS and LIM-OD fundus (B) after moderation at 16 weeks. Microaneurysms are indicated by white arrowheads. **(C-D)** Quantification of relative photoreceptor nuclei counts in the ONL from FDM-OD (C) and LIM-OD (D) groups normalized to the CT-OS group. (n = 3/group). **(E-F)** Quantification of acellular capillaries (E) and endothelial/perivascular (E/P) cell ratios (F) in PAS-stained, trypsin-digested retinas from CT-OS and FDM-OD groups. (n = 6/group). **(G-H)** Quantification of acellular capillaries (G) and E/P cell ratios (H) in PAS-stained, trypsin-digested retinas from CT-OS and LIM-OD groups. (n = 6/group). **(I-J)** Quantification of Evans Blue fluorescence intensity from retinal flatmount images in CT-OS and FDM-OD eyes (I), and in CT-OS and LIM-OD eyes (J). (n = 9/group). Results expressed as mean ± SEM. **p* < 0.05. *P* values were determined by unpaired two-tailed student’s t test (C-J).

**
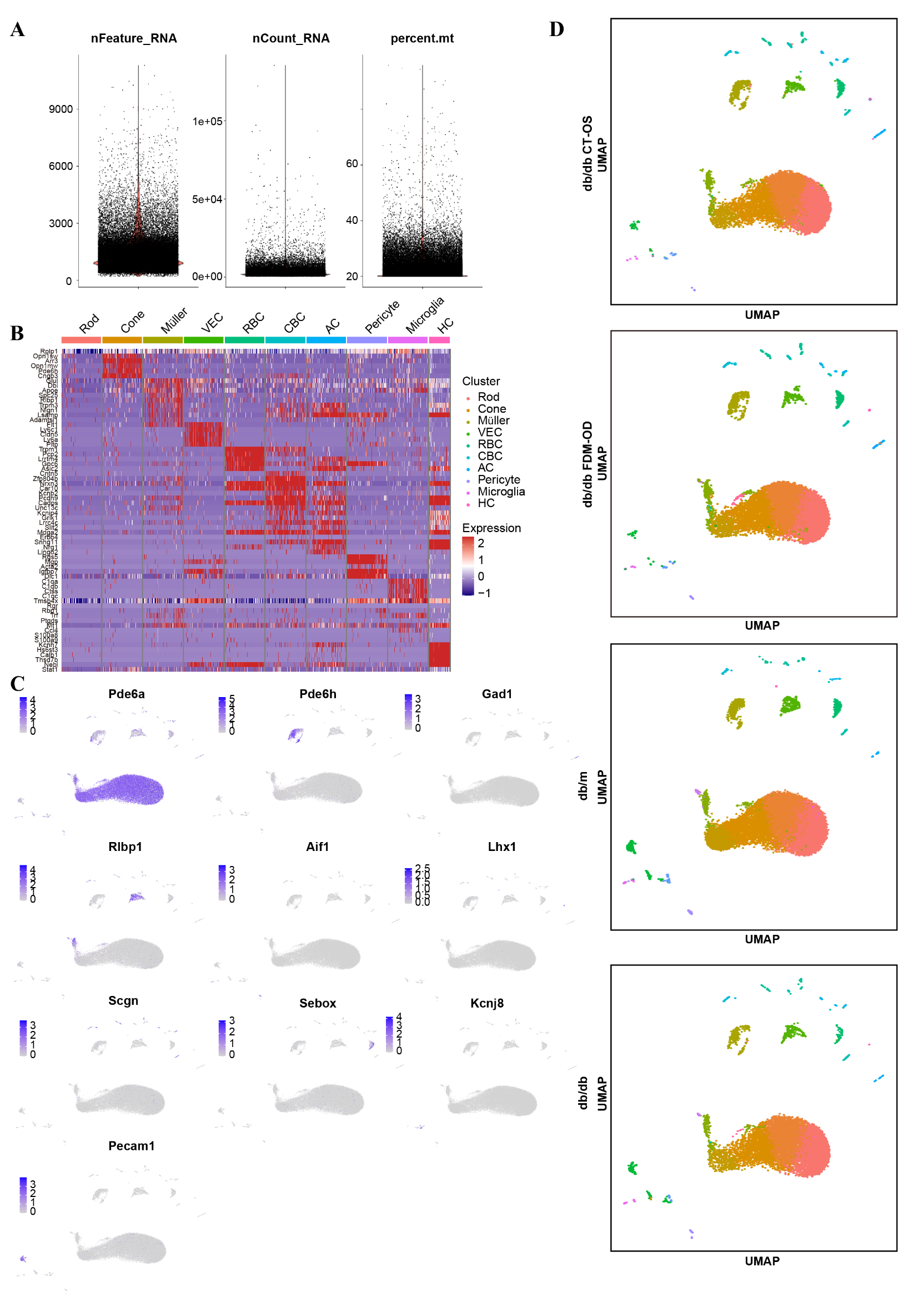
Supplementary Figure 3 Marker-based classification of retinal cell populations in single-cell RNA-seq data. (A)** Quality control metrics of scRNA-seq data. Violin plots show the distribution of the number of detected genes per cell (nFeature_RNA), total RNA counts per cell (nCount_RNA), and the percentage of mitochondrial gene content (percent.mt) across all cells prior to filtering. These metrics were used to assess and exclude low-quality cells. **(B)** Heatmap showing scaled expression of discriminative gene sets for major cell types in retina from all mice groups. **(C)** Feature plots displaying the expression of representative marker genes for each major retinal cell type/subtype on the UMAP embedding. The intensity of the color corresponds to the normalized expression level of each gene in individual cells. **(D)** Group-wise UMAP projections displaying the distribution of cells from each group (db/db FDM-OD, db/db CT-OS, db/db and db/m) across the identified clusters.


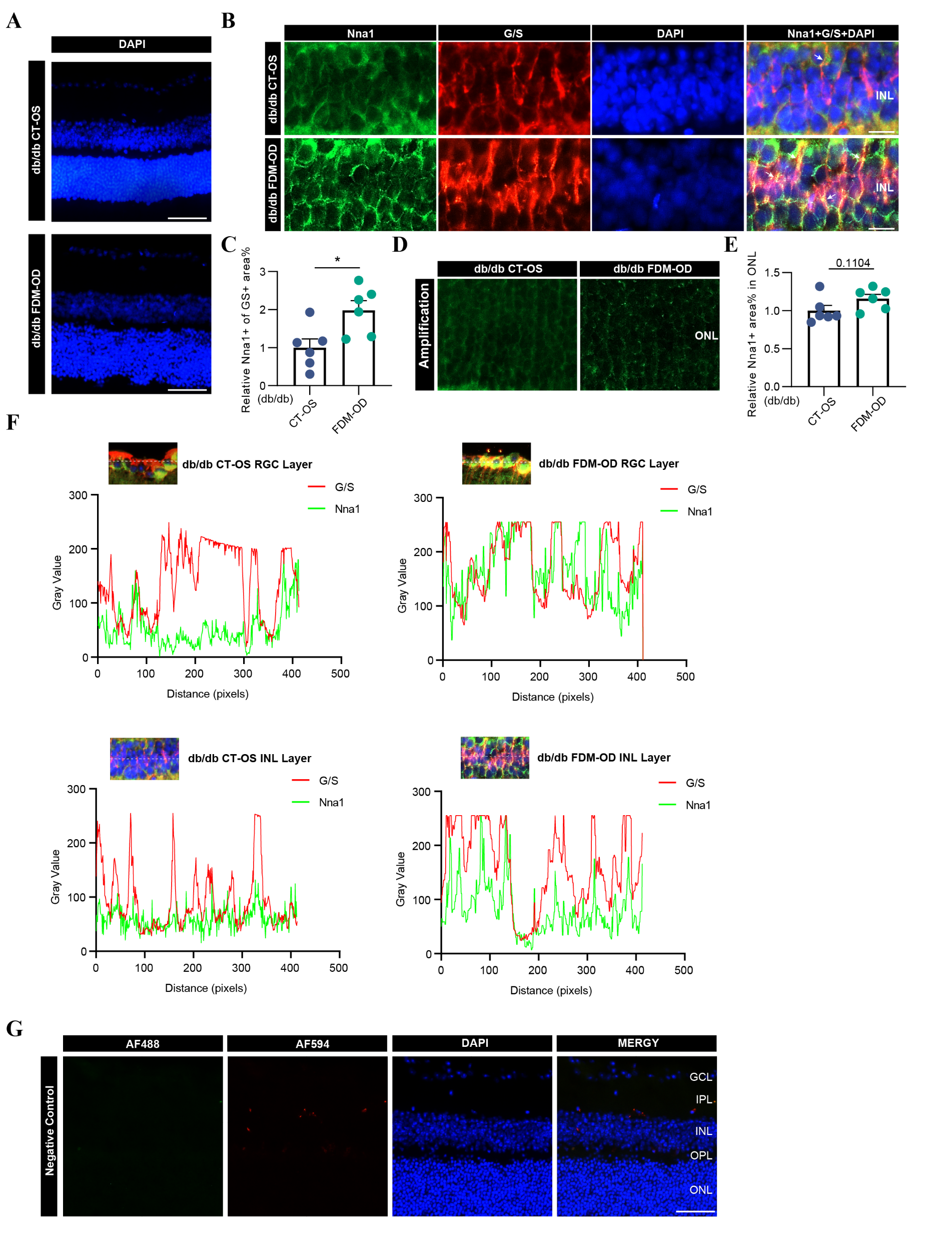


**Supplementary Figure 4 Colocalization analysis of Nna1 with Müller glia in db/db FDM-OD and CT-OS group. (A)** Retinal sections from db/db CT-OS and FDM-OD eyes were stained with Nna1 (green), G/S (red), and DAPI (blue). Only the DAPI channel is shown in the supplementary panel, as the full merged image is presented in the main figure. Scale bar indicate 50 μm. (n = 6/group). **(B)** Enlarged images corresponding to Figure 3J, stained with Nna1 (green), G/S (red), and DAPI (blue) in INL Layer. Scale bar indicate 10 μm. **(C)** Quantification of the relative Nna1^+^ area as a percentage of the G/S^+^ Müller cell area in db/db CT-OS and FDM-OD retinas. **(D-E)** Magnified views of the outer nuclear layer (ONL) from db/db CT-OS and FDM-OD retinal sections, together with quantification of the relative Nna1⁺ area. **(F)** Quantitative analysis of Nna1 (green) and G/S (red) colocalization in db/db CT-OS and FDM-OD retinas at RGC and INL layers, with significantly increased colocalization observed in db/db FDM-OD compared with CT-OS across both layers. **(G)** Negative control images of retinal sections stained with secondary antibodies (AF488: green and AF594: red) and DAPI. Scale bar indicate 50 μm. (n = 3/group). Results expressed as mean ± SEM. **p* < 0.05. *P* values were determined by unpaired two-tailed student’s t test (C, E).

**
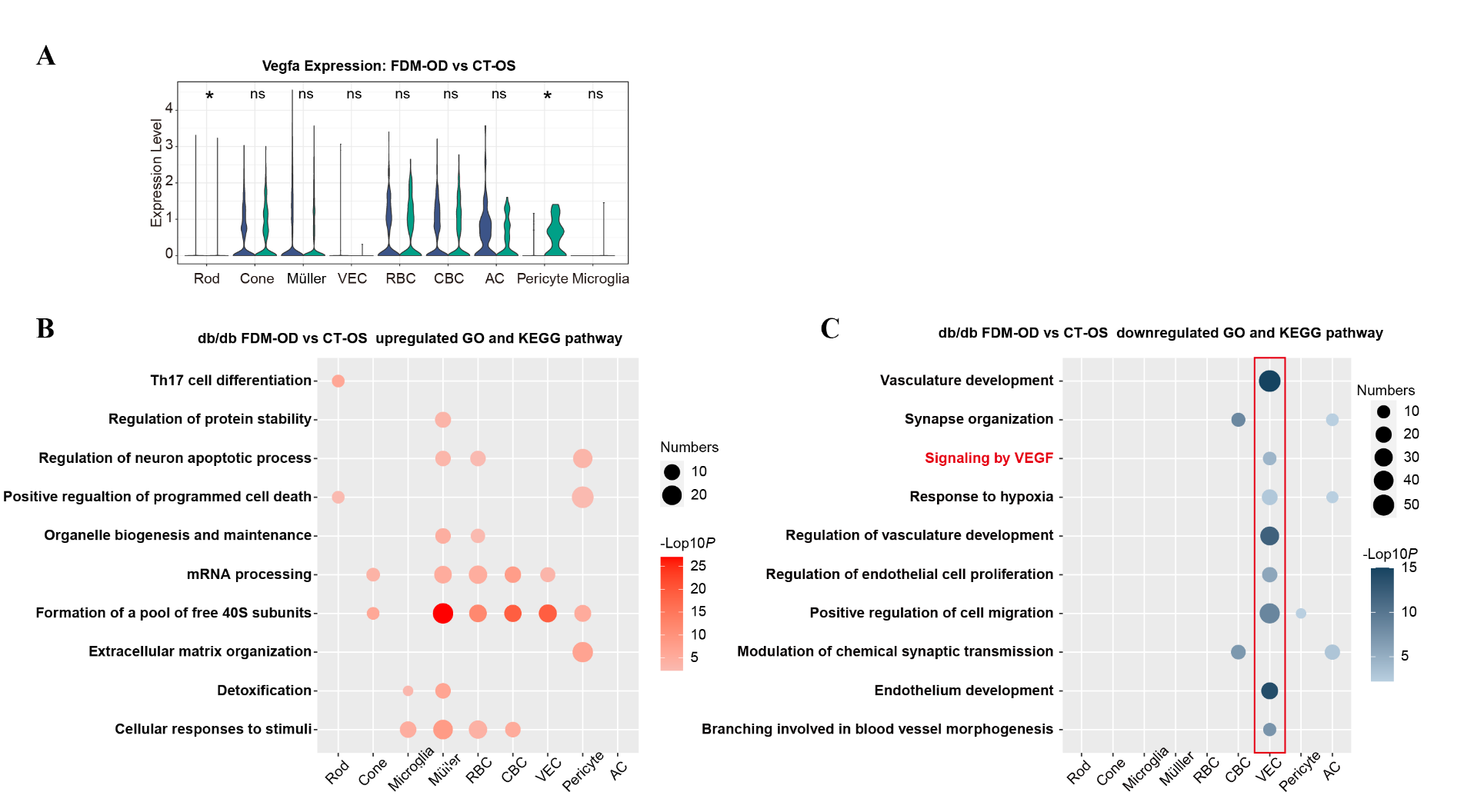
**

**Supplementary Figure 5 Differential gene expression and pathway changes across retinal cell types in db/db FDM-OD compared to CT-OS eyes based on scRNA-seq. (A)** Violin plots of VEGFA expression in retina subsets of db/db FDM-OD and CT-OS groups. **(B-C)** Bubble plot displaying representative enriched GO terms and KEGG pathway in upregulated DEGs (B) and downregulated DEGs (C) of retina subsets between db/db FDM-OD and CT-OS groups. **p* < 0.05. *P* values were determined by two-tailed Wilcoxon rank-sum test (A).


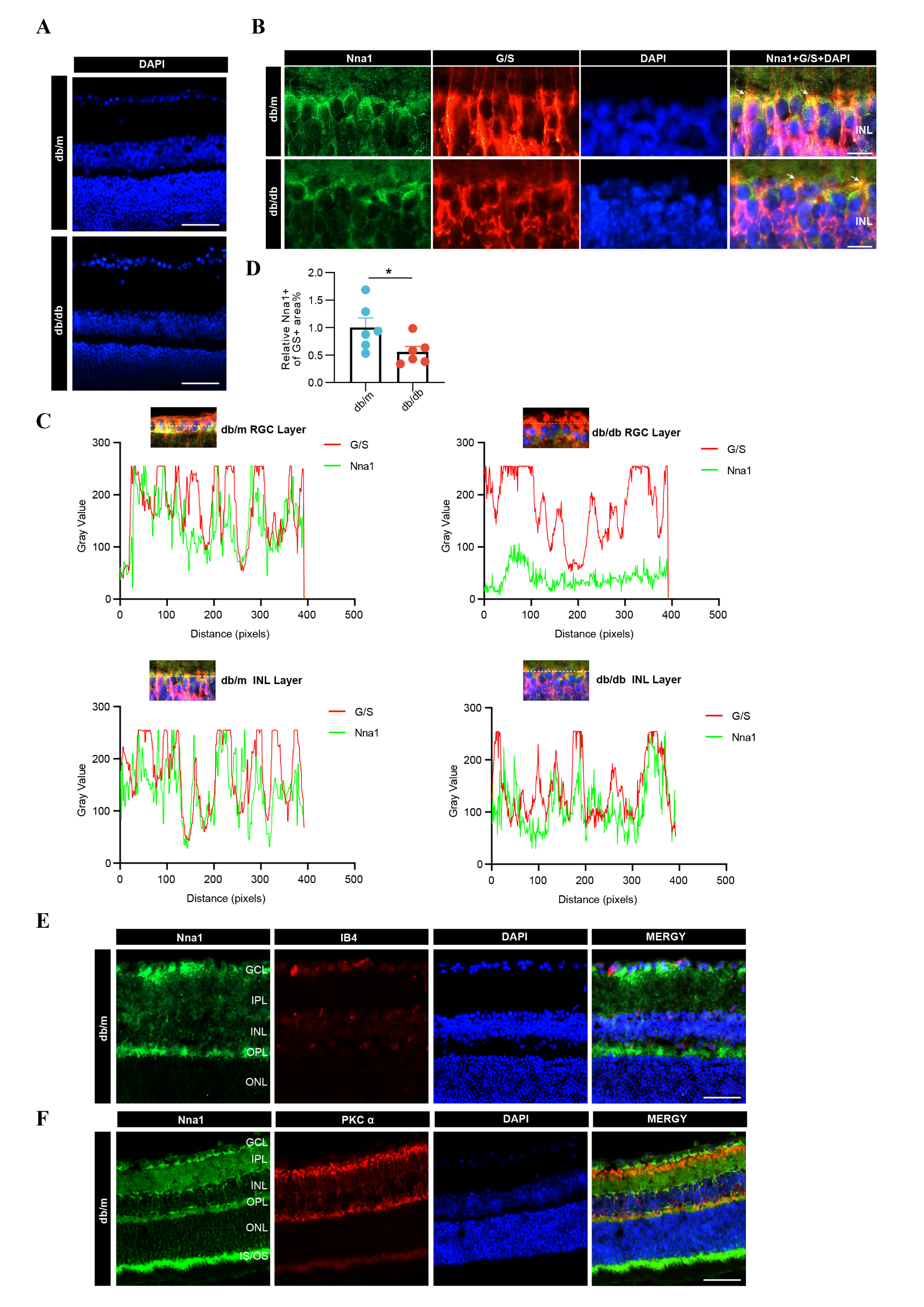


**Supplementary Figure 6 Colocalization analysis of Nna1 with Müller glia in db/db and db/m groups and Double immunofluorescence staining of Nna1 with vascular and bipolar cell markers. (A)** Retinal sections from db/db and db/m eyes were stained with Nna1 (green), G/S (red), and DAPI (blue). Only the DAPI channel is shown in the supplementary panel, as the full merged image is presented in the main figure. Scale bar indicate 50 μm. (n = 3/group). **(B)** Enlarged images corresponding to Figure 4F, stained with Nna1 (green), G/S (red), and DAPI (blue) in INL Layer. Scale bar indicate 10 μm. **(C)** Quantitative analysis of Nna1 (green) and G/S (red) colocalization in db/db and db/m retinas at RGC and INL layers, with significantly decreased colocalization observed in db/db compared with db/m across both layers. **(D)** Quantification of the relative Nna1^+^ area as a percentage of the G/S^+^ Müller cell area in db/db and db/m retinas. (n = 6/group). **(E-F)** Retina sections of db/m mice were processed for immunofluorescence using Nna1 (green) antibodies, IB4 (red) (E) or PKC α (red) (F) and DAPI (Blue). Scale bars indicate 50 μm. Results expressed as mean ± SEM. **p* < 0.05. *P* values were determined by unpaired two-tailed student’s t test (D).


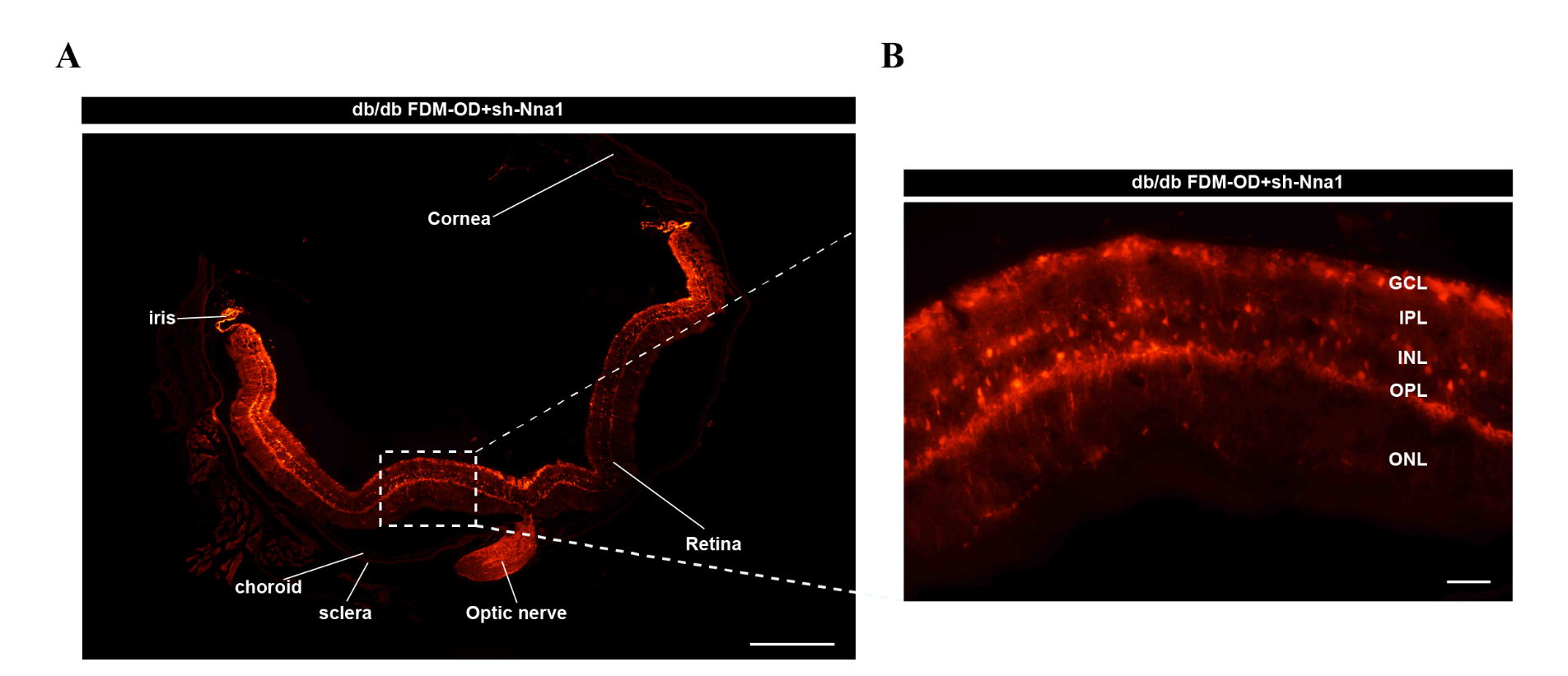


**Supplementary Figure 7 Validation of shNna1-AAV transduction efficiency. (A-B)** Representative fluorescence images showing efficient retinal transduction following intravitreal injection of shNna1-AAV carrying an mscarlet-reporter. Scale bars indicate 500 μm and 100 μm.
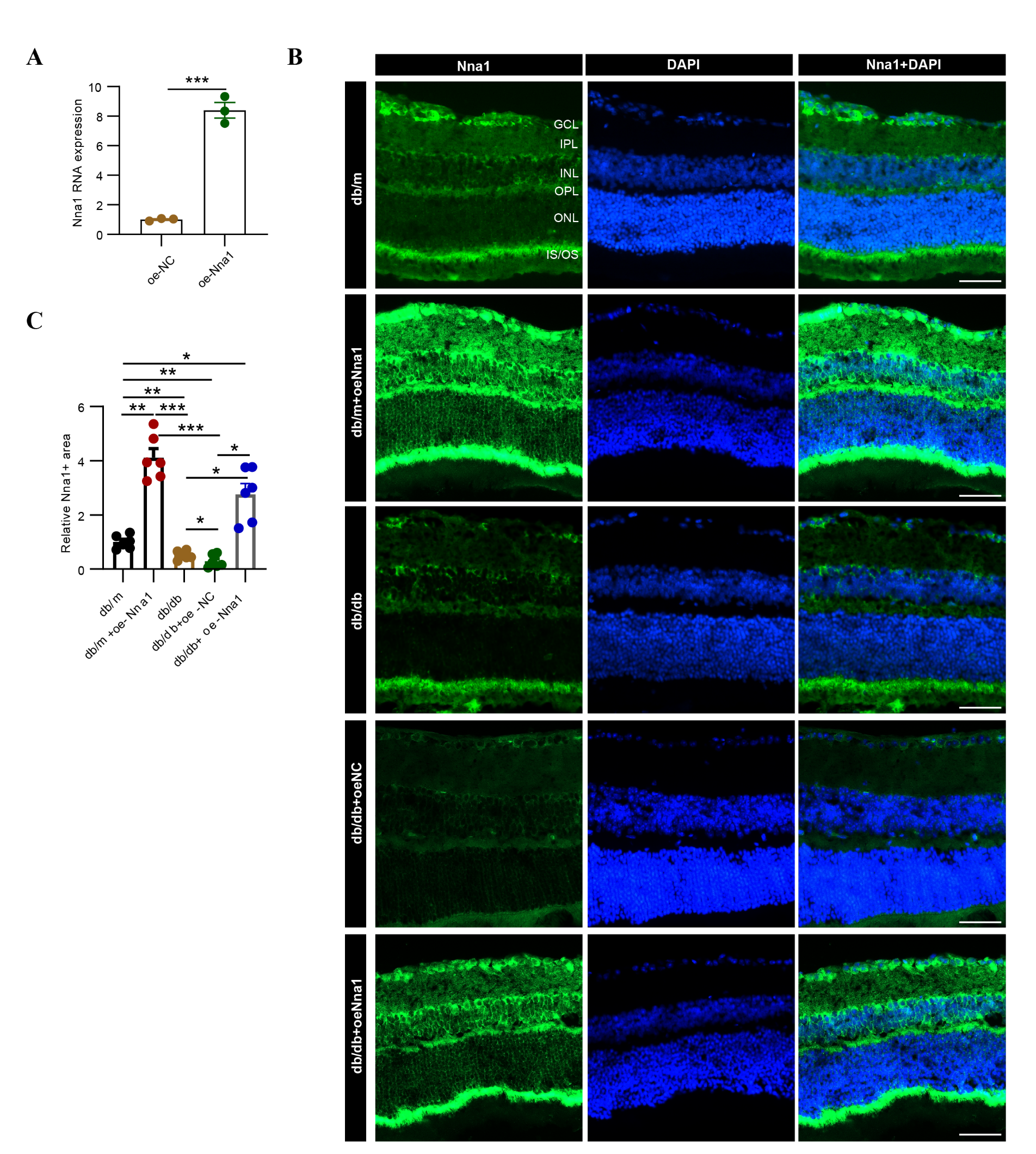


**Supplementary Figure 8 Immunofluorescence staining of retinal Nna1 expression following lentiviral-mediated overexpression. (A)** Quantification of the expression of Nna1 in db/db+oe-NC and db/db+oe-Nna1 retinas measured by RT-QPCR. (n = 3/group). **(B-C)** Immunofluorescence staining of Nna1 (green) and DAPI (blue) in retinal frozen sections from five experimental groups (db/m, db/m+oe-Nna1, db/db, db/db+oe-NC, db/db+oe-Nna1) after lentiviral-mediated overexpression. Scale bar indicate 50 μm. (n = 6/group). Results expressed as mean ± SEM. **p* < 0.05, ***p* < 0.01, ****p* < 0.001. *P* values were determined by unpaired two-tailed student’s t test (A) or one-way ANOVA (C).

**
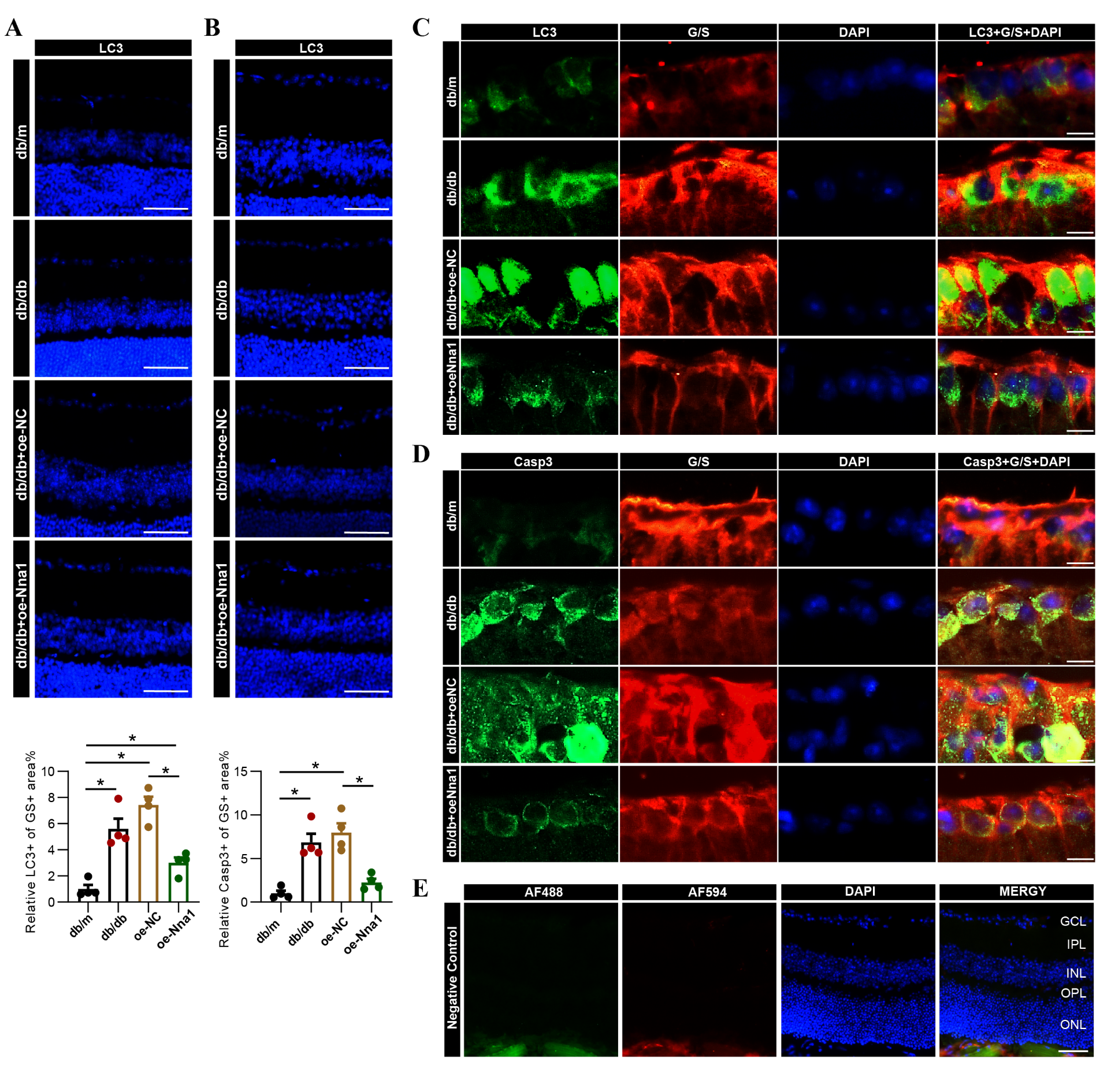
Supplementary Figure 9 Enlarged views and the negative results of immunofluorescence images shown in Figure 6E–F. (A-B)** Retinal sections from db/m, db/db, db/db+oe-NC, and db/db+oe-Nna1 mice, eyes were stained with LC3 (green) (A), CASP3 (green) (B), G/S (red), and DAPI (blue). Only the DAPI channel is shown in the supplementary panel, as the full merged image is presented in the main figure. Scale bar indicate 50 μm. (n = 3/group). **(C)** Enlarged images corresponding to Figure 6E, stained with LC3 (green), G/S (red) and DAPI (blue). Scale bar indicate 10 μm. (n = 3/group). **(D)** Enlarged images corresponding to Figure 6F, stained with Caspase-3 (green), G/S (red) and DAPI (blue). Scale bars indicate 10 μm. (n = 3/group). **(E)** Negative control images of retinal sections stained with secondary antibodies (AF488: green and AF594: red) and DAPI. Scale bar indicate 50 μm. (n = 3/group). Results expressed as mean ± SEM. **p* < 0.05. *P* values were determined by one-way ANOVA (C-D).


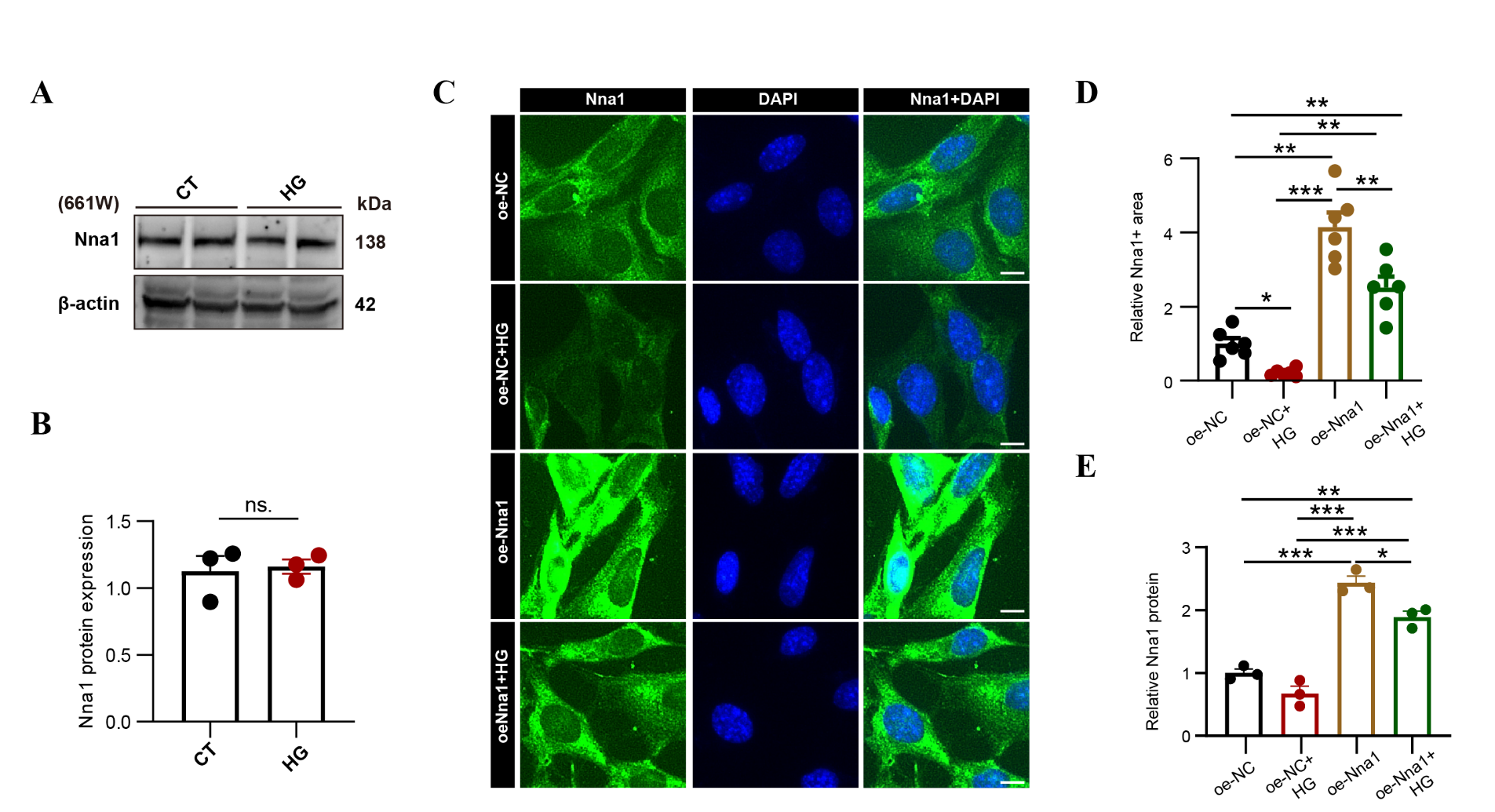


**Supplementary Figure 10 High glucose reduces Nna1 expression in rMC-1 cells, which is reversed by overexpression. (A-B)** Western blots images showing the protein level of Nna1 in 661W cells treated with HG or untreated (CT). (n = 3/group). **(C-D)** Immunofluorescence images of rMC-1 cells transfected with oe-NC or oe-Nna1 with or without HG treatment. Cells were stained with Nna1 (green), and DAPI (Blue). Scale bars indicate 10 μm. (n = 6/group). **(E)** Quantification of Nna1 protein expression in rMC-1 cells treated with HG or untreated, and transfected with oe-NC or oe-Nna1. (n = 3/group). Results expressed as mean ± SEM. **p* < 0.05, ***p* < 0.01, ****p* < 0.001. *P* values were determined by unpaired two-tailed student’s t test (B) or one-way ANOVA (D, E).


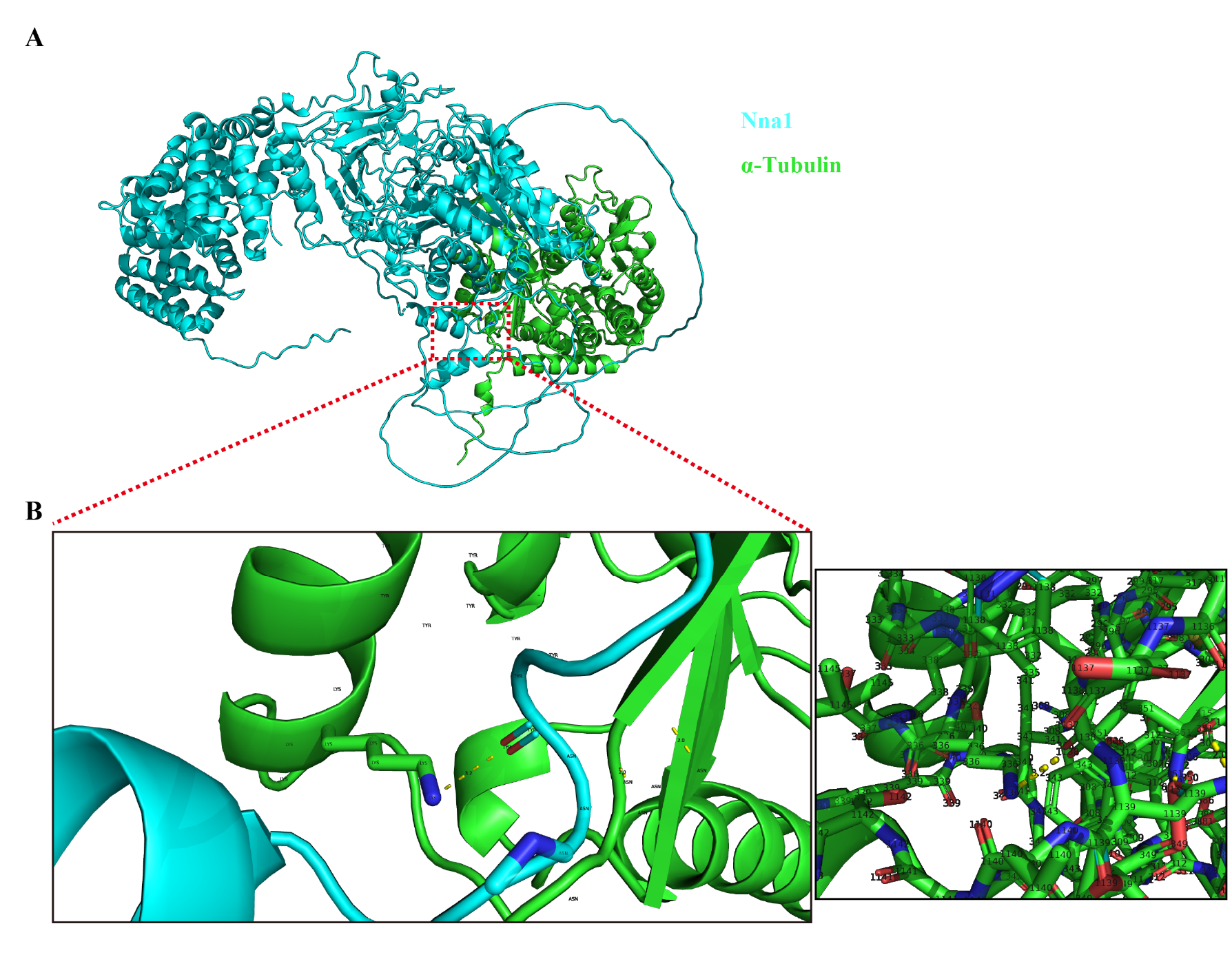


**Supplementary Figure 11 Predicted molecular docking model between Nna1 and α-tubulin. (A)** Predicted docking model of Nna1 (blue) and α-tubulin (green) generated using the ZDOCK server and visualized in PyMOL. **(B)** Enlarged view showing the predicted binding interface between Nna1 and α-tubulin.

**
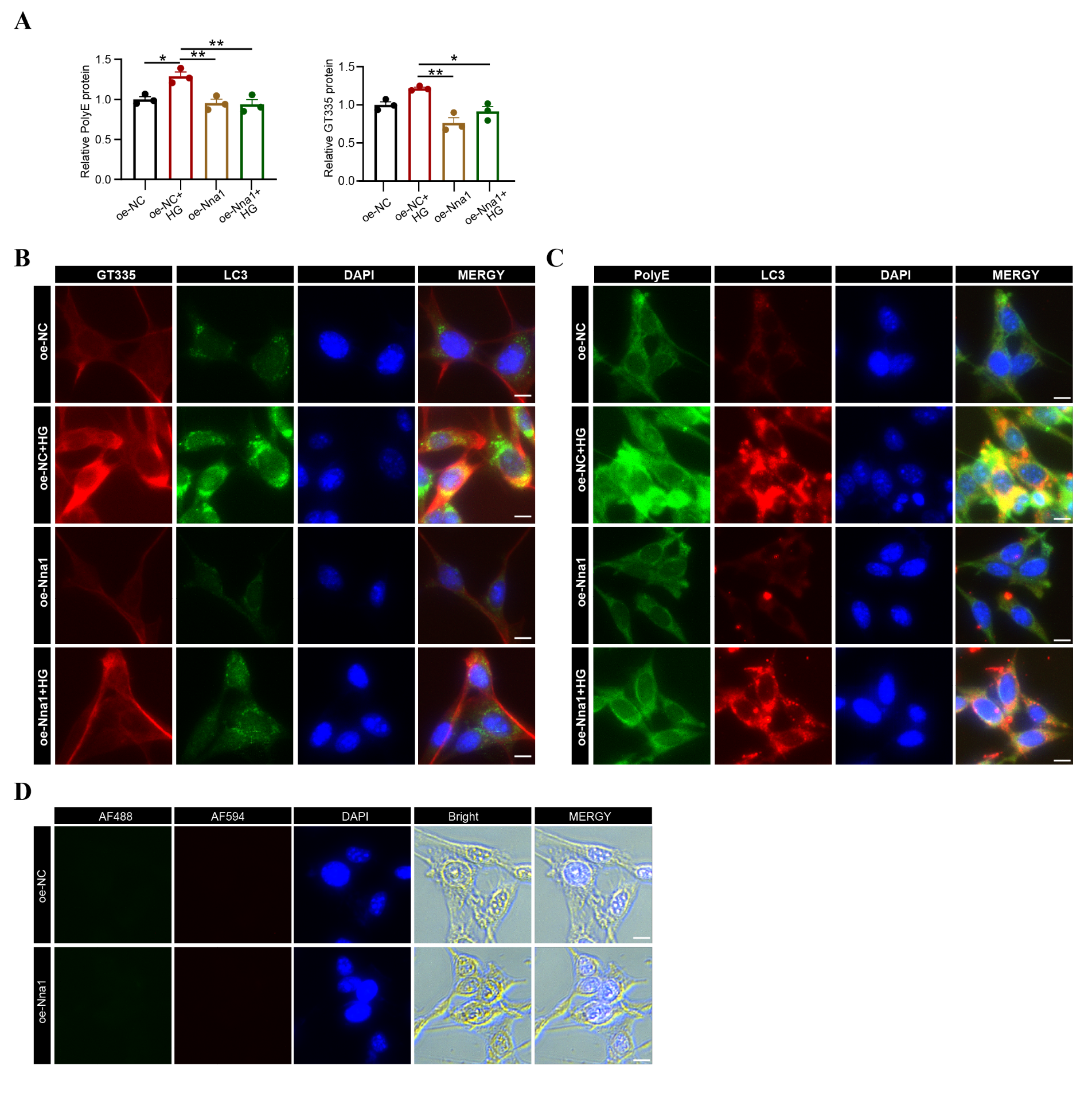
Supplementary Figure 12 LC3 colocalization with GT335 and PolyE in rMC-1 cells. (A)** Quantification of PolyE and GT335 protein expression in rMC-1 cells treated with HG or untreated, and transfected with oe-NC or oe-Nna1. (n = 3/group). **(B)** Immunofluorescence images of rMC-1 cells transfected with oe-NC or oe-Nna1 with or without HG treatment. Cells were stained with GT335 (red), LC3 (green) and DAPI (Blue). Scale bars indicate 10 μm. (n = 3/group). **(C)** Immunofluorescence images of rMC-1 cells transfected with oe-NC or oe-Nna1 with or without HG treatment. Cells were stained with PolyE (green), LC3 (red) and DAPI (Blue). Scale bars indicate 10 μm. (n = 3/group). **(D)** Negative control images of rMC-1 cells stained with secondary antibodies (AF488: green and AF594: red) and DAPI (blue). Scale bars indicate 10 μm. (n = 3/group). Results expressed as mean ± SEM. **p* < 0.05, ***p* < 0.01. *P* values were determined by one-way ANOVA (A).
