# Supplementary material for: Single‐Cell RNA Sequencing of Retina Reveals Nna1 Upregulation in Myopic Diabetic Retinopathy as a Protective Factor Against Diabetic Damage
Source: Adv Sci (Weinh). 2025 Nov 5;13(5):e00438. doi: 10.1002/advs.202500438 (PMC12849878; doi:10.1002/advs.202500438)

Figure 2

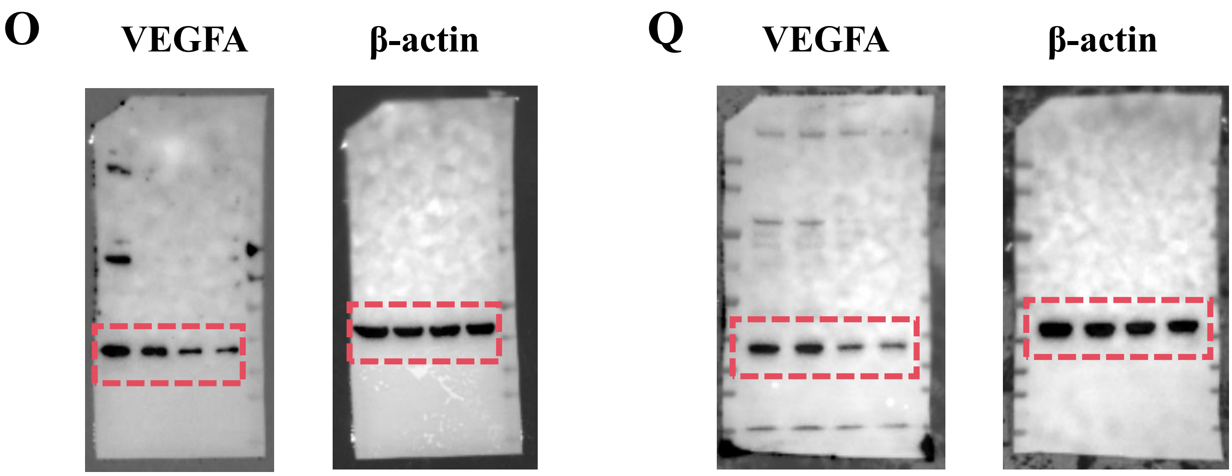

Figure 3

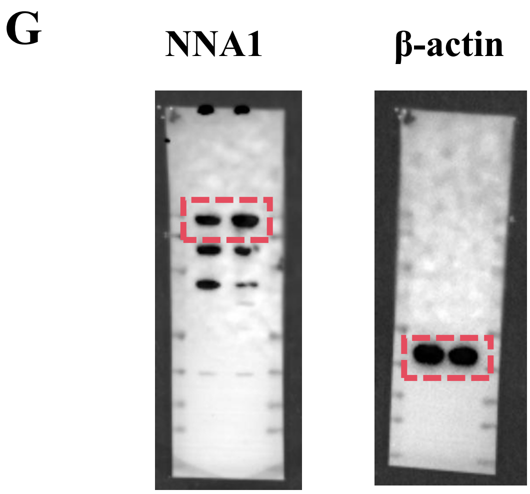

Figure 4

C

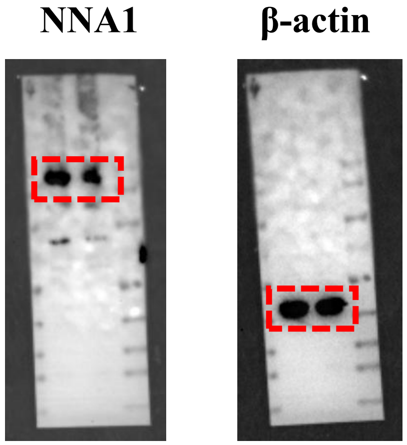

G

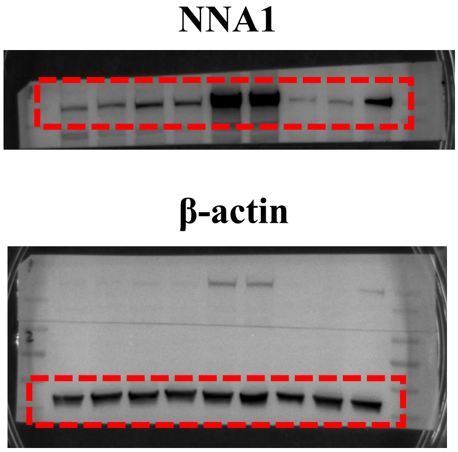

H

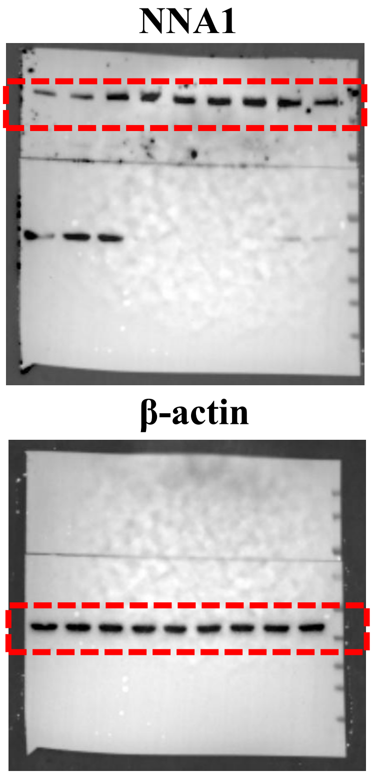

O

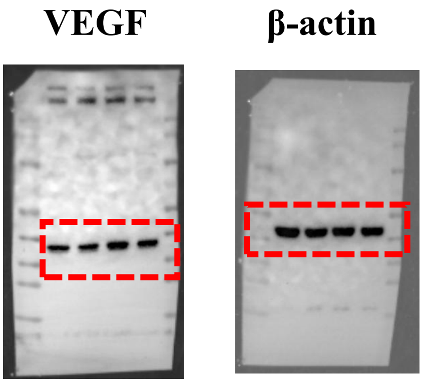

Figure 5

A

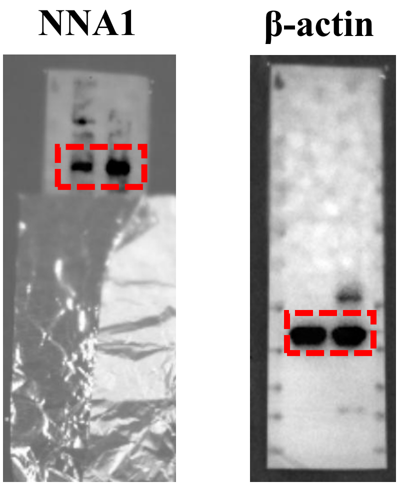

Figure 6

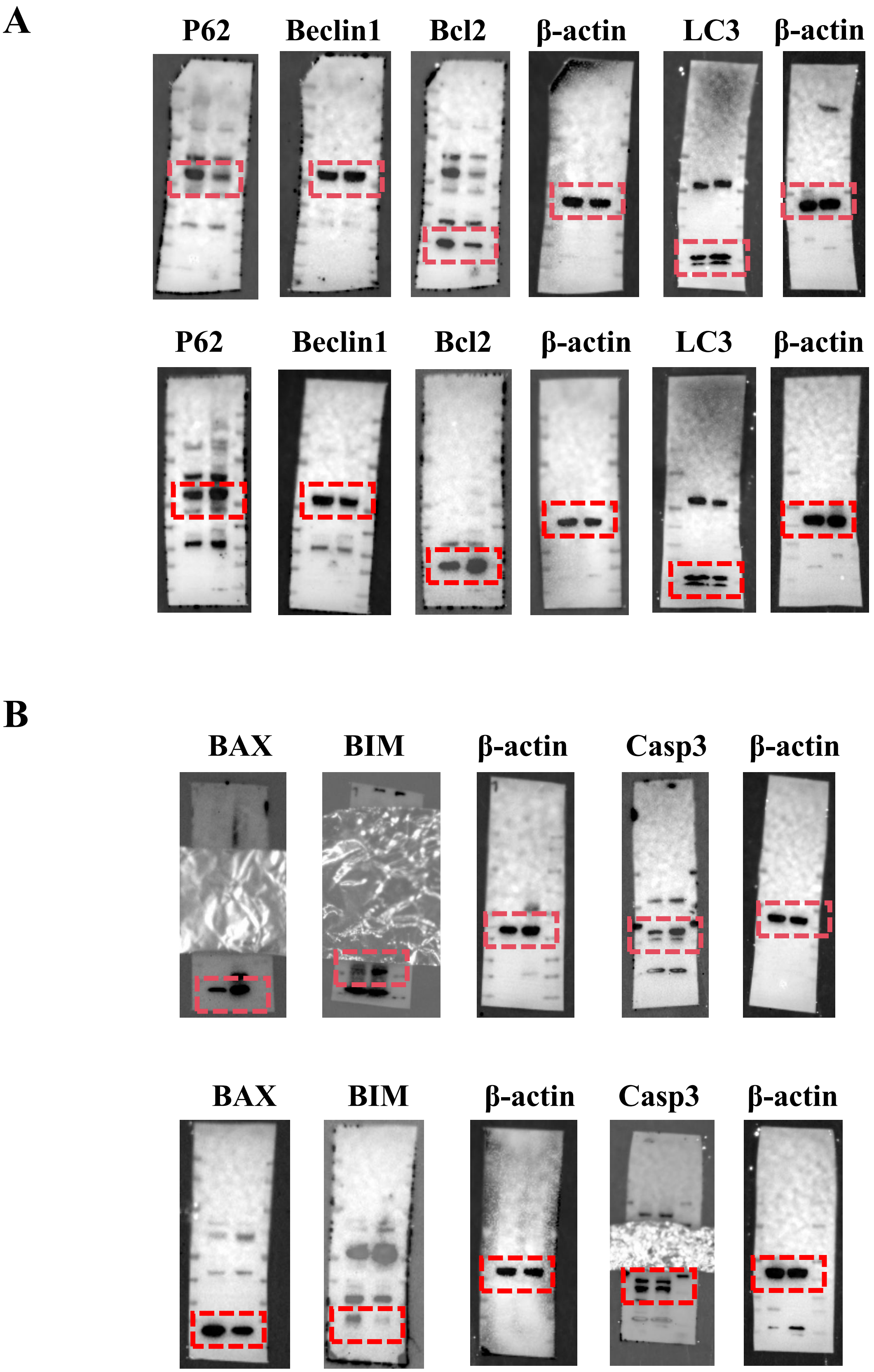
A
P62
Beclin1
Bcl2
β-actin
LC3
β-actin
P62
Beclin1
Bcl2
β-actin
LC3
β-actin
B
BAX
BIM
β-actin
Casp3
β-actin
BAX
BIM
β-actin
Casp3
β-actin

Figure 7

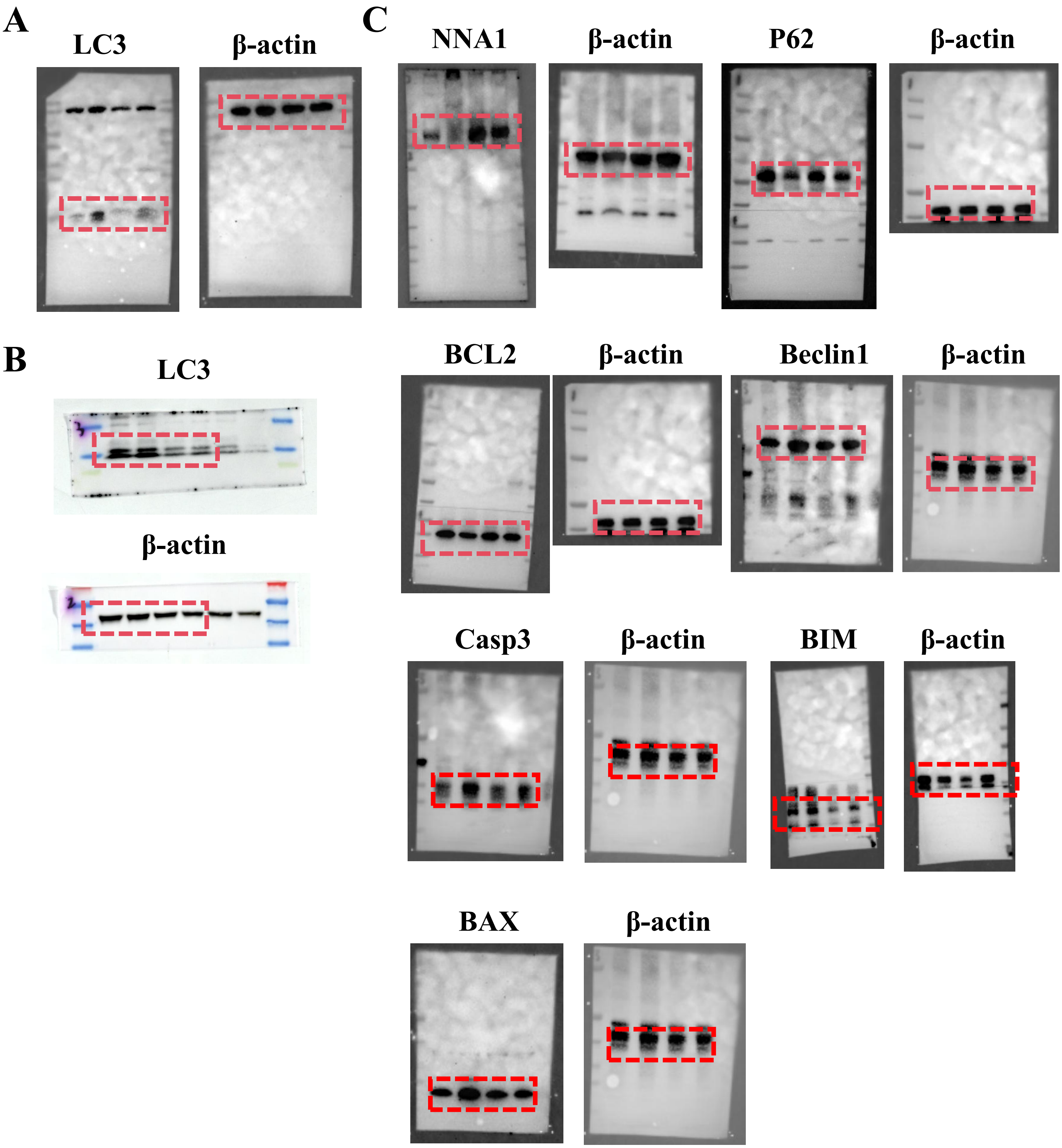
A
LC3
β-actin
B
LC3
β-actin
C
NNA1
β-actin
P62
β-actin
BCL2
β-actin
Beclin1
β-actin
Casp3
β-actin
BIM
β-actin
BAX
β-actin

Figure 7

I

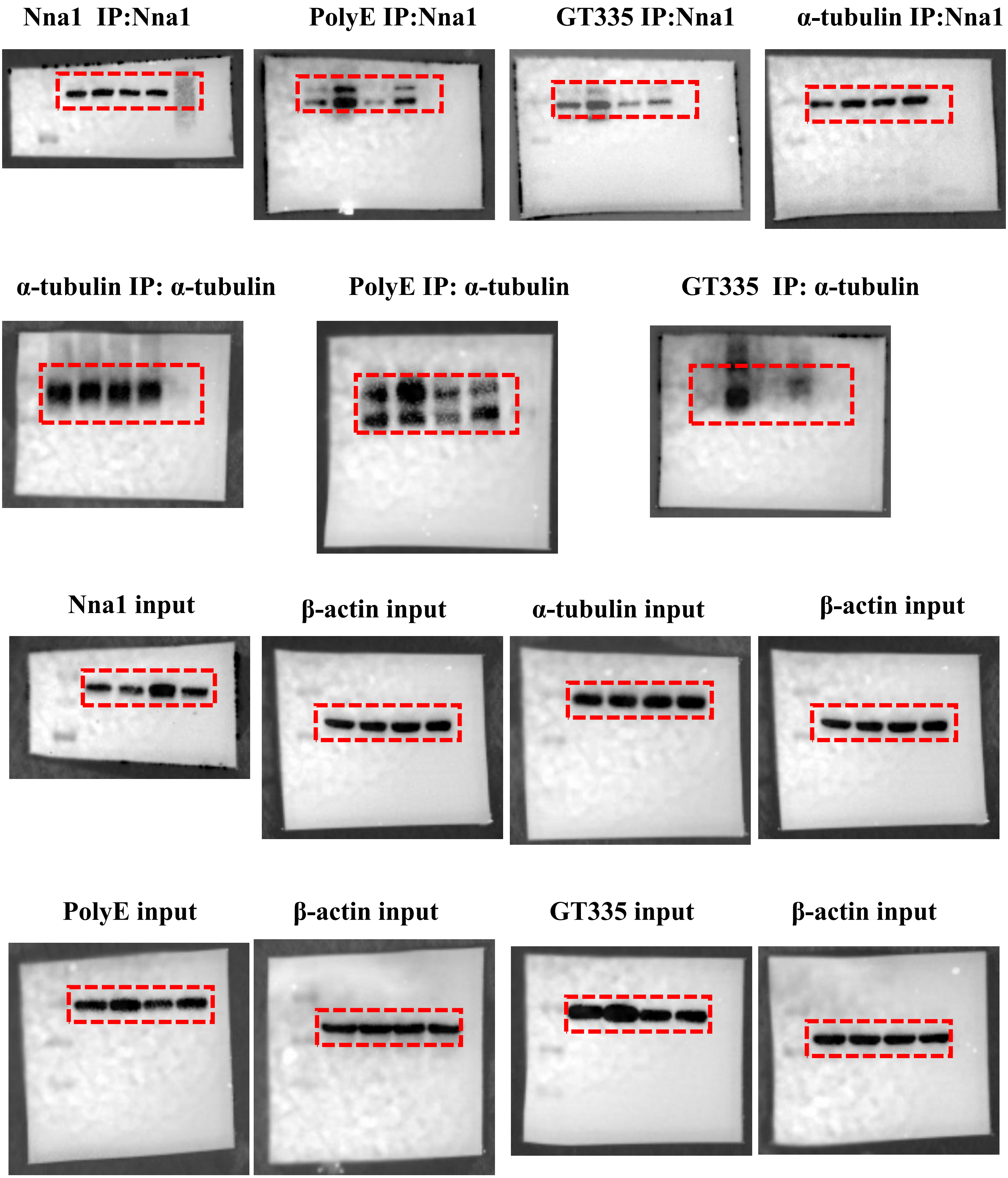
Nna1 IP:Nna1
PolyE IP:Nna1
GT335 IP:Nna1
α-tubulin IP:Nna1
α-tubulin IP: α-tubulin
PolyE IP: α-tubulin
GT335 IP: α-tubulin
Nna1 input
β-actin input
α-tubulin input
β-actin input
PolyE input
β-actin input
GT335 input
β-actin input

Figure S10

A

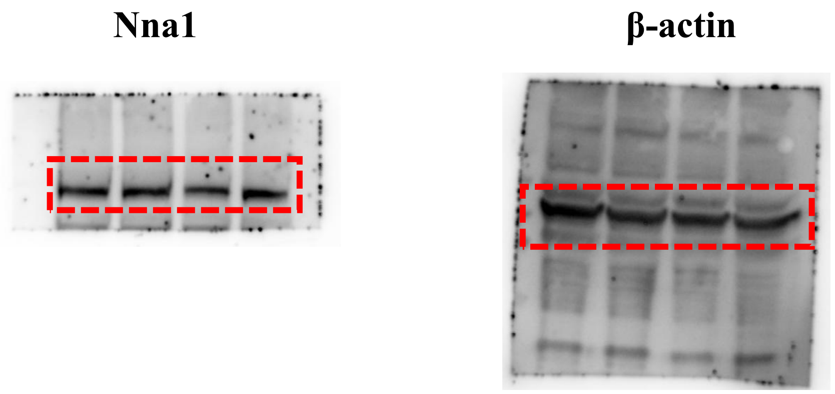

Supplement: Supplementary file 3 — Supporting Information [file ADVS-13-e00438-s002.pdf]
